# Effect of Real-World Perturbations on Wave Breaking due to a Sharp-Crested Superharmonic Instability

**DOI:** 10.1007/s42286-025-00116-7

**Published:** 2025-03-20

**Authors:** A. Mansar, M. R. Turner, T. J. Bridges, F. Dias

**Affiliations:** 1 Centre Borelli, École Normale Supérieure Paris-Saclay, 91190 Gif-sur-Yvette, France; 2 School of Mathematics and Physics, University of Surrey, Guildford, Surrey, GU2 7XH England; 3 School of Mathematics and Statistics, University College Dublin, Belfield, Dublin 4, Ireland

**Keywords:** Unstable, Dipole, Stokes wave

## Abstract

The mechanism for the emergence of breaking water waves in deep water, based on the superharmonic instability of periodic Stokes waves, is tested for the effect of real-world perturbations (dissipation, approximation error, changes in depth, non-zero air density, fluctuations in wave and frame speed). An implicit perturbation is added to a large-amplitude unstable Stokes wave, which is then taken as initial data in a direct numerical solution of the Navier–Stokes equations, using the Basilisk numerical software package. An SVD-based filtering algorithm is used to extract the shape of the unstable wave that grows on the background Stokes wave. We find a dipole shape in the filtered wave that correlates with the superharmonic unstable mode. Our findings show that the inclusion of real-world effects has little qualitative effect, when they are kept small, on the emergence of breaking. We conclude that the mechanism of crest instability of Stokes waves leading to wave breaking is a robust mechanism that is likely to occur in nature.

## Introduction

One of the first theoretical explanations for the breaking of water waves in deep water was based on the superharmonic instability, which arises when a travelling Stokes wave has a sufficiently large amplitude. In the superharmonic unstable regime, the form of the eigenfunction has a dipole shape that is concentrated around the crest. This shape, when added to the basic state, lowers the amplitude in front of the crest and raises it behind, creating a mechanism for overtopping. Unstable large-amplitude solitary waves are also susceptible to a similar wave-breaking mechanism.

**Fig. 1:**
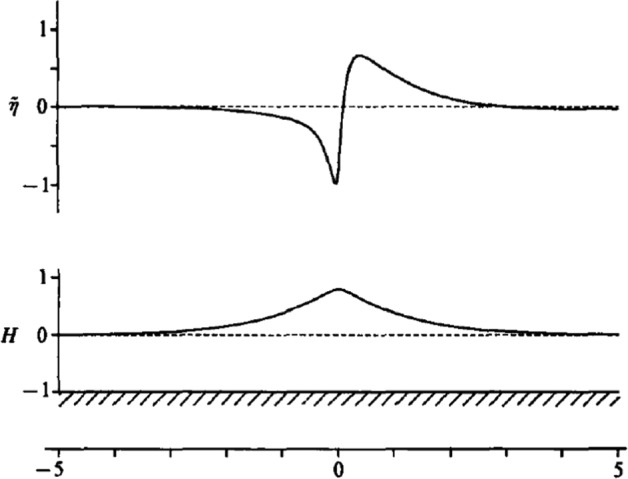
The initial data for direct numerical simulation in Tanaka et al. [54] where a superharmonic unstable eigenfunction are added to a basic solitary wave. This figure is reproduced from Figure 1 of Ref. [54]

This scenario was first confirmed in the seminal paper of Tanaka et al. [54]. They integrated the initial-value problem for two-dimensional inviscid water waves, with initial data taken to be a large-amplitude unstable solitary wave, perturbed by the addition of the unstable eigenfunction. Most of the amplitude of the unstable eigenfunction is concentrated around the crest, having a dipole-like shape. Figure 1, reproduced from Ref. [54], shows a schematic of the initial data used for their direct numerical simulations. The amplitude of the basic solitary wave is of $\mathcal {O}(1)$ and the amplitude of the perturbation is $\mathcal {O}\big (10^{-2}\big )$.

**Fig. 2:**
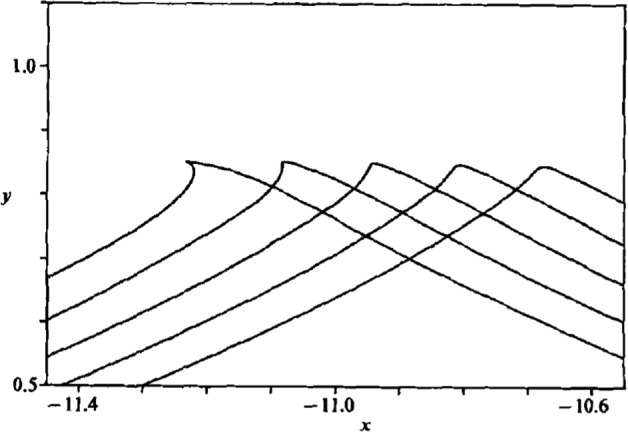
Integration of the initial-value problem for a solitary wave perturbed by a crest instability as depicted in Fig. 1. This figure is reproduced from Figure 5 of Ref. [54]

With the initial data as in Fig. 1, the initial-value problem is integrated forward in time. The direction of travel of the wave is right to left. Adding the dipole-shaped eigenfunction to the solitary wave decreases the overall amplitude ahead of the crest, and enhances the overall amplitude behind the crest. The combined action creates an overtopping effect that grows during the time integration. One example of this form of wave breaking is shown for a sequence of times in Fig. 2, reproduced from Ref. [54]. The growth of the crest instability and overtopping is clearly visible.

The initial-value problem in Ref. [54] is integrated using a boundary-integral method based on the inviscid and irrotational Euler equations (the details of the numerical algorithm are in Dold [17]).

In this paper, we focus on the superharmonic instability of large-amplitude *periodic* Stokes travelling waves. The terminology was coined by Longuet-Higgins in the seminal paper [36]. The distinguishing feature of this instability is that it is *co-propagating*; that is, it has the same wavelength and speed as the Stokes wave. This feature is to be contrasted with subharmonic instabilities and the Benjamin–Feir instability where the perturbation has a different wavelength and speed. For the case of unstable periodic Stokes waves, the above wave-breaking scenario was first observed in a paper of Longuet-Higgins and Cokelet [38], then later in the numerical simulations of Jillians [31], with the most complete results to date in the paper of Longuet-Higgins and Dommermuth [39].

The question we address in this paper is how robust is this mechanism to real-world conditions? To get as close to real-world conditions as possible we study the initial-value problem via direct numerical simulation of the Navier–Stokes equations using the Basilisk software package [26]. In this package, a Stokes wave is not an exact solution. Therefore, we take a Stokes periodic travelling wave—in the superharmonic unstable region of parameter space—as initial data. Since it is not an exact solution, there is an implicit perturbation due to the approximation error. Hence, *a priori* we do not know the shape of the perturbation and how it correlates with the dipole shape of the unstable eigenfunction. Note that Grue [23] suggests that the third-order Stokes wave provides a better approximation for the kinematics of steep wave events observed in laboratory wave tanks compared to fully nonlinear wave solutions. Regarding energy dissipation, Deike et al. [15] indicate that the third-order solution aligns well with experimental data. Here, we aim to strike a balance between the third-order solution, which accurately describes real-world events, and a highly nonlinear solution (5th order) that includes higher harmonics, crucial for capturing the superharmonic instability. This trade-off allows us to achieve both the practical accuracy needed to represent real-world phenomena and the theoretical depth required to account for complex nonlinear interactions.

To address the correlation issue, we first calculate the exact (to numerical approximation) eigenfunctions associated with the superharmonic unstable Stokes waves in §3. Secondly, we develop a filtering algorithm, using a singular value decomposition (SVD), to decompose the perturbation into modes. We then identify the modes that have a dipole structure and correlate with the exact eigenfunction. It is these modes that are responsible for the overtopping leading to breaking. In addition to dissipation, the Basilisk algorithm has a non-zero upper fluid density, non-zero surface tension, and approximates infinite depth by a very large depth. Moreover, the Stokes wave itself is approximate, and by embedding it in Basilisk it changes speed. Remarkably, we find that the addition of all these perturbations, as long as they are kept small, do not prevent the wave breaking mechanism due to superharmonic instability, which is based predominantly on the dipole shape of the perturbation.

An outline of the paper is as follows. The theory of the superharmonic instability has been developed in several directions, including other water-wave contexts, and its implication for wave breaking has also been studied. In Sect. 2, we give a review of past work in this area. In Sect. 3, the eigenfunctions associated with an unstable superharmonic mode are here calculated afresh as they are needed for comparison purposes in the direct numerical simulation. In Sect. 4, we present the numerical strategy for solving the initial-value problem for the full Navier–Stokes equations with viscosity, based on the Basilisk algorithm. Results on integration using Basilisk are discussed in Sect. 5. A summary and future directions are discussed in Sect. 6.

## Literature Review

The superharmonic (SH) instability of periodic travelling Stokes waves was first discovered in numerical calculations of Longuet-Higgins [36], some 10 years after the discovery of the Benjamin–Feir (BF) instability. In the case of BF, the instability arises at low amplitudes, even for weakly nonlinear Stokes waves (as long as $kh > 1.363$), and the unstable eigenfunction is “modulated”, that is, the wavelength differs from the wavelength of the Stokes wave. On the other hand, SH instability does not arise until the Stokes wave is of sufficiently high amplitude, and the wavelength of the unstable SH eigenfunction is the same as the basic Stokes wave.

**Fig. 3:**
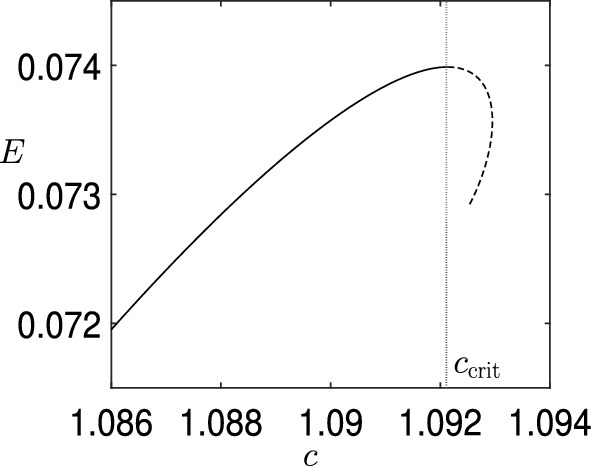
A plot of the energy versus wave speed for a Stokes periodic travelling wave, computed using the algorithm in Sect. 3.1. The solid line represents SH stable solutions, while the dashed line represents SH unstable solutions

A few years later, Tanaka [52] set about to repeat the results of Longuet-Higgins on the SH instability. He confirmed the results both qualitatively and quantitatively, and also discovered something new. He found that as the amplitude of the Stokes wave increased, the primary SH instability arose at precisely the point where the curve of energy versus wave speed reached a maximum. A plot of the energy versus wave speed along a branch of Stokes waves, newly computed using the algorithm of Sect. 3.1, is shown in Fig. 3.

Figure 3 is interesting for several reasons. First, it is clear that the highest Stokes wave is not the wave of highest energy, since the amplitude continues to increase after the energy-speed maximum. Second, Tanaka’s calculations in Refs. [52, 53] show that the SH instability arises at the maximum, and so the branch to the right of the maximum is SH unstable. The solid curve in the figure represents SH stable waves, but they may be unstable to other perturbations. Third, the diagram in Fig. 3 shows that for energy values just below the maximum there are two distinct periodic Stokes waves with the same energy: a slow wave and a fast wave, with the slow wave SH stable and the fast wave SH unstable. These two waves will be important in the time integration of the initial-value problem.

The connection between the energy maximum and the SH instability was confirmed numerically by Longuet-Higgins [37]. Shortly thereafter, Saffman [49] proved that a maximum in a plot of the energy versus wave speed corresponds precisely to a transition of SH stability to instability. This result was further refined by taking Saffman’s theory to the next order in amplitude in Bridges [6], and the Hamiltonian structure was used to give a more general result, with application to SH instability of Stokes waves with vorticity in Sato and Yamada [50].

Longuet-Higgins and Tanaka [41] were the first to discover that as the branch of periodic Stokes wave is continued to higher amplitude, a second point of SH instability change occurs. They show that this is also true in the SH instability of solitary waves. The implication in Fig. 3 is that the curve will have a vertical tangency at some higher amplitude of the Stokes wave, and then a minimum at a yet higher value. In Ref. [41] only two such points were found. These results have been taken to the next level by Deconinck et al. [13], finding several more points numerically and using asymptotic theory to predict that there is likely to be an infinite number of new SH unstable eigenvalues emerging at each extrema of the energy. Korotkevich et al. [35] go further in finding another class of instabilities, also with real eigenvalues, but the eigenvalues are associated with eigenfunctions that have double the wavelength of the basic Stokes wave. These two instabilities alternate in importance as the highest wave is approached. In either case, the eigenfunctions that dominate the dynamics are localised near the crest of the wave.

**Fig. 4:**
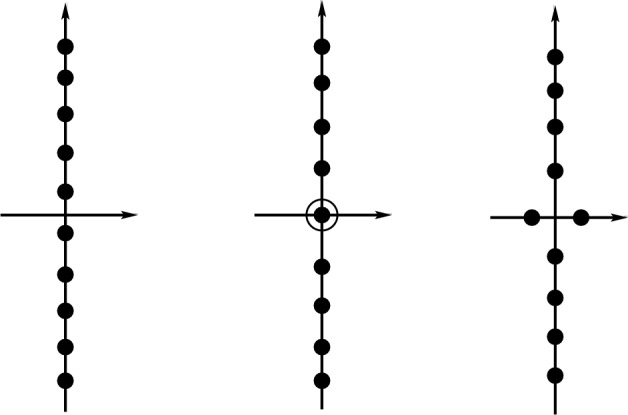
Schematic of the eigenvalues in the linearization about a Stokes waves with superharmonic perturbations. The primary SH mode transition from stable to unstable (or vice versa) passing through zero, and the SH modes with non-zero frequency are assumed to be purely imaginary

The linearization about a Stokes wave with superharmonic perturbations has an infinite number of eigenvalues. A schematic is shown in Fig. 4. In addition to the primary SH instability, associated with a zero eigenvalue transitioning from a purely imaginary pair to a purely real pair, there is a countable set of modes with non-zero frequency. Some of the higher frequency modes can be unstable, but in this paper, we assume the configuration shown in Fig. 4 where all the higher modes are stable and on the imaginary axis. When we refer to a “second point of SH instability” as above, we are referring to the primary unstable mode at different parameter values.

Adding surface tension to the problem results in travelling waves that can differ quite significantly from the Stokes periodic wave. The exact solution of Crapper [11] shows that the highest wave may not even be single-valued, leading to uniformly travelling waves that are overhanging (see Ref. [11] and its citation trail). Hogan [25] was the first to undertake a linear stability analysis, for SH modes, of the Crapper waves at high amplitudes (at low amplitudes, where the wave height is a single-valued function, results on linear stability were reported by Chen and Saffman [10]). Hogan finds SH unstable modes, but at higher frequencies. The primary SH instability associated with a maximum of the energy is absent for Crapper waves. Indeed, calculations of Hogan [24] show that the energy is a monotone function of the wave speed. Explicit formulas for $c^2$ and energy are given on page 176 of Ref. [24] from which one can infer that *dE*/*dc* is never zero. The absence of the primary SH instability associated with $E'(c)=0$, for Crapper waves, is confirmed by Tiron and Choi [55]. However, recalculation of the higher frequency SH instabilities in Ref. [55] found that the SH modes were stable for all values of the amplitude, right up to the maximum, in contrast to Ref. [25]. Tiron and Choi attributed this discrepancy to the need for higher precision calculations, and a disagreement in how the Krein signature is calculated in Ref. [25].

Restricting to infinite depth, and the primary SH mode, the above results on the role of capillarity indicate that gravity waves, with small surface tension, will be SH unstable at large amplitude. However, if the surface tension dominates gravity, the SH instability will disappear. This latter observation will be important in numerical simulations, if the Bond number (ratio of the gravity forces to surface tension forces), which is a variable parameter in Basilisk, is made too small, the mechanism for wave breaking may disappear.

**Fig. 5:**
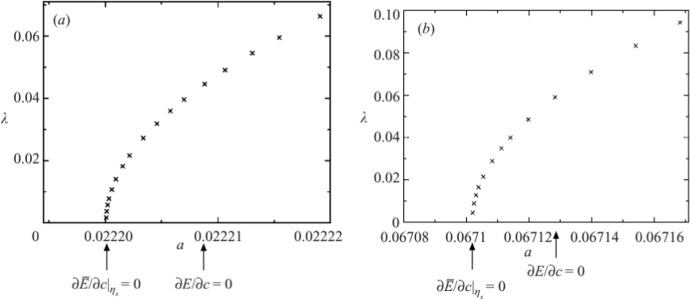
Plot reproduced from Figure 1 of Kataoka [33] showing that the SH instability change does not occur at the energy maximum $\partial E/\partial c=0$, but is influenced by the mass integral ${\overline{\eta }}$

Adding finite-depth changes the picture. Continuing the above thread on capillary waves, Blyth and Parau [4] study the linear stability of SH modes for the finite-depth version of Crapper waves (Kinnersley waves). They found values of parameters where SH instability does occur. Hence, there will be a critical value of the depth, for each value of the surface tension, below which SH instability can be expected. On the other hand, pure gravity waves travelling in finite depth were first studied, with respect to SH modes, by Zufiria and Saffman [58], and then more comprehensively by Kataoka [33]. Both papers show that an SH instability occurs at large amplitude. An interesting feature, discovered by Kataoka, is that the change of SH instability no longer occurs at the maximum of the energy, but is shifted by an amount determined by the mass integral. The mass integral only comes into play at finite depth. Figure 5, reproduced from Ref. [33], shows definitively that the behavioural change of the SH instability is shifted away from the energy maximum.

The effect of vorticity on the superharmonic instability of periodic travelling waves, in infinite depth, has been studied by Blyth and Parau [5]. They add a background shear flow to the periodic travelling wave, and set gravity to zero. Stability is studied analytically for small amplitude, and numerically for large amplitude. The main conclusion is that instability is shown to occur for any non-zero amplitude wave of this type. A Hamiltonian-based theory for SH instability of travelling waves with non-zero vorticity is developed in Sato and Yamada [50], for the case of finite and infinite depth. Their theory shows that, for fixed values of the vorticity, the SH stability transition still occurs at the maximum of the energy. However, they do not present any results, and their analysis of the finite-depth case does not agree with Kataoka [33].

On the other hand a special case of travelling waves with vorticity is periodic travelling waves near the Kelvin–Helmholtz instability, where the velocity profile is a Heaviside function. In this case, the SH instability occurs already for weakly nonlinear waves and it can be obtained analytically. This latter result is given in Benjamin and Bridges [1], and it is also confirmed analytically there that the change of stability is synchronised with the maximum of the energy, $E'(c)=0$. In Ref. [1], the fluid depth is infinite in both the lower and upper layers. In the absence of shear, but still interfacial waves, the SH instability is not found at low amplitudes, but is found at high amplitude, by Kataoka [34], for the pure gravity case. Again the fluid depth is infinite in the lower and upper layers. He points out that mathematically the SH instability result for two layer flow is similar to the one layer pure gravity case, showing moreover that the change of stability is synchronised with the change of the sign of $E'(c)$.

### Wave Breaking

The idea that an unstable periodic travelling wave could lead to wave breaking was first pointed out by Longuet-Higgins and Cokelet [38]. They integrated the equations for the inviscid and irrotational water-wave problem and found that SH unstable Stokes waves would overturn and break. However, their results were limited to a few examples without a clear understanding of the mechanism involved. The two main papers on the connection between SH instability and wave breaking are Tanaka et al. [54] and Longuet-Higgins and Dommermuth [39], the former for a solitary wave basic state, and the latter for a periodic basic state.

A key issue that was first observed in the paper [54] is the importance of the “right sign” in the initial data. The wave height perturbation is of the form

2.1 $$\begin{aligned} \eta (x,t) = H(x-ct) + \delta {\widetilde{\eta }}(x-ct,t)\,, \end{aligned}$$

with $H(x-ct)$ the uniformly travelling solitary wave, and ${\widetilde{\eta }}(x-ct,t)$ the eigenfunction associated with the unstable SH mode, both relative to a frame of reference moving at speed *c*. This expression with $t=0$ is used to initialise the time integration.

The small parameter $\delta$ can be of either sign. In Ref. [54], it was found that with $\delta =-0.01$ all initial data led to wave breaking. On the other hand, when taking $\delta =+0.01$ the evolution was bounded, and indeed, the solution was attracted to a slower solitary wave, with the same energy, but lower amplitude. This scenario is consistent with the energy viewpoint as shown in Fig. 3. For each unstable solitary wave, there is a stable solitary wave, of lower amplitude, at the same energy. It appears that when $\delta =+0.01$, the faster unstable solitary wave is attracted to the slower wave, whereas when $\delta =-0.01$ the faster solitary wave is outside the basin of attraction of the slower solitary wave, and becomes more nonlinear instead, and with no other solution to be attracted to, it generates an infinite slope in the wave height and then overturns. Some analysis justifying both scenarios is in §4.2 of Ref. [54].

The second paper to study the initial-value problem for the full water-wave equations, with initial data including a SH unstable mode, is the paper of Jillians [31]. His paper was also the first to consider a periodic basic state. His results are similar to those found for unstable solitary waves by Tanaka et al. [54] in the case of $\delta =-0.01$. He uses the form (2.1) with $H(x-ct)$ a periodic Stokes wave which is SH unstable. For negative $\delta$, he finds the initial value problem evolves into a breaking wave. On the other hand, the case of positive $\delta$ is inconclusive. Overall the results of Jillians are incomplete. In their commentary, Longuet-Higgins and Dommermuth [39] speculate that the calculations of Jillians have lower precision, and the value of $|\delta |$ is greater, thereby missing the basin of attraction.

**Fig. 6:**
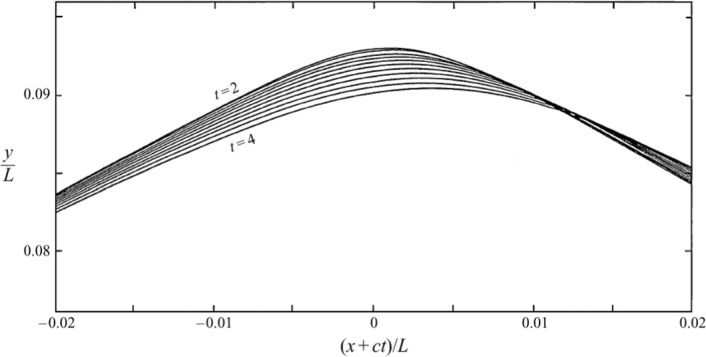
Plot reproduced from Figure 11 of Ref. [39] showing a negative perturbation leading to the wave settling down to a slower wavetrain of the same energy

In the calculations of Longuet-Higgins and Dommermuth [39], a higher accuracy was used and a scenario that is closer to Ref. [54] was found. In their formulation, the sign of $\delta$ is reversed. Hence, in their case, positive $\delta$ corresponds to the breaking case, and negative $\delta$ corresponds to the case where the perturbation is attracted to the slower periodic travelling wave at the same energy. They used more recent calculations of the SH unstable eigenvalues and their eigenfunctions due to Longuet-Higgins and Tanaka [41]. In the case of negative $\delta$, [39] find a result similar to Ref. [54], where the solution is attracted to a slower periodic travelling wave. This scenario is shown in Fig. 6 which reproduces Figure 11 of Ref. [39].

**Fig. 7:**
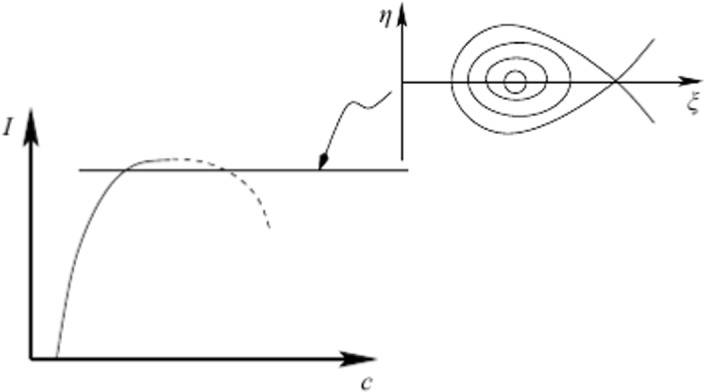
Schematic of the recurrence phenomena observed in the numerics of Ref. [39]. The left depicts the energy (or momentum) versus wave speed showing the curve of periodic travelling waves, while the right picture shows a schematic of a projection onto a plane of the phase-space. This figure is reproduced from Figure 3 of Ref. [6]

They also continue the integration in time and find a form of recurrence; that is, after a sufficiently long time, the solution cycles back to the neighbourhood of the unstable periodic travelling wave. This scenario is consistent with a homoclinic orbit as shown schematically in Fig. 7 which is reproduced from Figure 3 of Ref. [6].

**Fig. 8:**
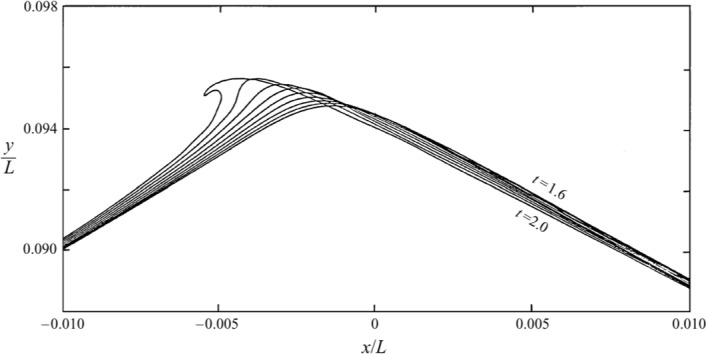
A microbreaker emerging from the superposition of a periodic travelling wave and a SH unstable eigenfunction, reproduced from Figure 9 of Ref. [39]

Figure 7 is obtained by projecting the infinite-dimensional space onto a two-dimensional subspace in which there is a saddle and a centre, with a homoclinic orbit. In the infinite-dimensional space, the homoclinic orbit will not exactly close up, but the recurrence phenomena will still be evident. In Fig. 8, from Longuet-Higgins and Dommermuth [39], the development of a microbreaker emerging from a crest instability in the case of a positive perturbation is shown.

In summary, the evolution of an SH unstable basic state into a microbreaker is very similar in both the case of a solitary wave basic state and a periodic travelling wave.

#### Wavelength Doubling and Potential Wave Breaking

The dipole structure of the eigenfunction of the SH instability occurs in other instabilities, and may provide alternative scenarios for wave breaking. An example is the case of perturbations with twice the wavelength of the underlying Stokes wave. It is shown by Deconinck et al. [13] that if one chooses a Stokes wave with an energy value just below the maximum, the solution is stable with respect to the SH instability, but is unstable with respect to perturbations with any non-zero Floquet parameter. In the case of an unstable perturbation that has a wavelength that is twice the wavelength of the underlying Stokes wave, the part of the perturbation in one wavelength has a shape that corresponds to a dipole-like structure. Hence, unstable wavelength doubling provides another potential scenario for wave breaking, at a lower energy value.

#### SH Instability and Wave Breaking in the Whitham Equation

The connection between SH instability and wave breaking has also been found in the Whitham equation. In dimensionless form, the Whitham equation is given by

2.2 $$\begin{aligned} u_t + \mathcal {K}\star u_x + \frac{3}{2}uu_x = 0\,, \end{aligned}$$

where $\mathcal {K}$ is the kernel of a convolution operator defined in Fourier space by

2.3 $$\begin{aligned} \widehat{\mathcal {K}}(\kappa ) = \sqrt{\frac{\textrm{tanh}(\kappa )}{\kappa }}\,, \end{aligned}$$

and $\kappa$ is the wavenumber. *u*(*x*, *t*) represents the dimensionless surface displacement. This equation is Hamiltonian. In Carter et al. [8], periodic travelling waves of this equation are constructed and it is found that plots of the energy (Hamiltonian function) versus the wave speed have a maximum, and at this maximum there is a transition of SH instability. Remarkably, the eigenfunction of the unstable SH mode has precisely the dipole structure found in the full water-wave problem and described above. They then reproduce the Tanaka et al. [54] experiment, by perturbing the basic state with this dipole eigenfunction. A form of wave breaking emerges, but it is not of the form witnessed in the water-wave problem since the solutions of the Whitham equation are constrained to be single-valued functions of *x*. Nevertheless, the key features of SH instability, dipole structure, and a form of wave breaking do arise in the model equation. Further results of Carter [7] show that as the amplitude of the basic state is increased further, a second critical point of the energy is found with the emergence of a second SH unstable mode, again very similar to the water-wave problem as found in Ref. [41].

#### Summary

The key observations emerging from the above literature review are that, in both the case of periodic travelling waves and solitary waves, in two space dimensions, the SH instability produces an eigenfunction with a dipole structure, emphasising the crest, and called a crest instability, and addition of this eigenfunction as a small perturbation to the basic state, with the right sign, leads to the formation of a microbreaker. It appears to be sufficient that the perturbation amplitude is of the order of 1% of the amplitude of the basic state. The value of 1% is empirically chosen. Any small value will do, but too small a value will require a longer time to generate sufficient growth for breaking.

## Computation of Unstable Superharmonic Modes

The eigenfunctions associated with an unstable SH mode are newly calculated here as they are needed for compatibility with the Basilisk direct numerical simulations. In addition, the basic state will need to be computed in compatible coordinates, for feeding into the linear stability calculation. In two space dimensions, it is conventional to use a conformal mapping of the water-wave problem to compute Stokes waves, either using conformal mapping with the velocity potential and stream function as independent coordinates (e.g. [36]) or using general conformal mapping coordinates (e.g. [35]). However, the initial-value problem using Basilisk is in primitive coordinates, with (*t*, *x*) as independent variables and the velocity field as dependent variables. For compatibility, we work directly in these variables. To this end, we use a two-space-dimensional version of Okamura [45], which is formulated with velocity potential and free surface as dependent variables.

An independent Matlab code has been constructed to perform the calculations, following the theory of Okamura [45] for the basic state, and the two-dimensional version of the theory for the linear stability problem following Ioualalen and Kharif [27]. The results for the basic state have been validated against the numerical results presented in Ref. [52], and the results for the linear stability problem are validated against the calculations of Ref. [36].

### Computing the Basic State

The basic state is the classical non-dimensional two-dimensional periodic Stokes wave in infinite depth, nondimensionalised by lengthscale $k^{-1}$ and timescale $\omega ^{-1}$ where *k* is the wavenumber and $\omega$ the frequency of the incident wave. The non-dimensional spatial coordinates (*x*, *z*) are oriented so that *z* points vertically upwards and *x* is a horizontal variable in the direction of the travelling wave. The free surface of the wave is represented by the function single-valued $z=\eta (x,t)$, although an overhanging free surface can still emerge in the Basilisk numerical scheme.

The flow is assumed to be inviscid, incompressible and irrotational, with velocity potential $\phi (x,z,t)$ which satisfies the following set of equation:

3.1 $$\begin{aligned} \nabla ^2\phi= &   0\,,\quad \text{ for }\quad z<\eta (x,t)\,, \end{aligned}$$

3.2 $$\begin{aligned} \eta _t+\phi _x\eta _x-\phi _z= &   0\,,\quad \text{ on }\quad z=\eta (x,t)\,, \end{aligned}$$

3.3 $$\begin{aligned} \phi _t+\frac{1}{2}\nabla \phi \cdot \nabla \phi +\eta= &   Be\,,\quad \text{ on }\quad z=\eta (x,t)\,, \end{aligned}$$

3.4 $$\begin{aligned} \phi _z\rightarrow &   0\,,\quad \text{ as }\quad z\rightarrow -\infty \,, \end{aligned}$$

where *Be* is the Bernoulli constant. The wave propagates without change of shape, hence, in non-dimensional variables, we introduce the new variable

3.5 $$\begin{aligned} X=x-c t, \end{aligned}$$

where *c* is the phase speed of the wave which is determined as part of the solution. In *X*, *z* coordinates, the velocity potential satisfies

3.6 $$\begin{aligned} \phi _{XX}+\phi _{zz}=0~~~\textrm{for}~~~z<\eta (X), \end{aligned}$$

which is solved together with the dynamic boundary condition on the free surface

3.7 $$\begin{aligned} 0 = P(X,z):=-c\phi _X+\frac{1}{2}\left( \phi _X^2+\phi _z^2\right) +z~~~\textrm{on}~~z=\eta (X), \end{aligned}$$

and kinematic boundary condition

3.8 $$\begin{aligned} 0= &   Q(X,z):=c^2\phi _{XX}+\phi _X\left[ -2c\phi _{XX}+\phi _X\phi _{XX}+\phi _z\phi _{Xz}\right] ,\nonumber \\  &   +\phi _z\left[ -2c\phi _{Xz}+\phi _X\phi _{Xz}+\phi _z\phi _{zz}+1\right] \,,~~~\textrm{on}~~z=\eta (X), \end{aligned}$$

with the same bottom boundary condition (3.4).

Equation (3.8) is more complicated than the usual kinematic boundary condition (3.2), but is useful in dealing with basic flows near to the steepest wave where a sharp crest forms, as it does not contain any spatial derivatives of the free surface [44]. This version of the kinematic condition is equivalent to

3.9 $$\begin{aligned} \frac{DP}{Dt}=0~~~\textrm{on}~~z=\eta (X). \end{aligned}$$

The following periodicity and symmetry conditions are imposed on the velocity potential

$$\begin{aligned} \phi (X,z)=\phi (X+2\pi ,z),~~~~\textrm{and}~~~~\phi (X,z)=-\phi (-X,z)\,. \end{aligned}$$

We also introduce the wave steepness parameter

3.10 $$\begin{aligned} \epsilon =\frac{1}{2}\left[ \eta (0)-\eta (\pi )\right] , \end{aligned}$$

which is defined to be half the peak to trough height for the waves. The numerically obtained limiting steepness is $\epsilon = 0.4436$.

The algorithm proceeds by expressing the velocity potential in a finite Fourier series of the form

3.11 $$\begin{aligned} \phi (X,z)=\sum _{j=1}^{{\widetilde{N}}-1} A_j\sin (jX)\exp (j z)\,, \end{aligned}$$

where $A_j$ are unknown coefficients, which together with the phase speed *c* make up the ${\widetilde{N}}$ unknowns for the problem. The ${\widetilde{N}}$ unknowns are calculated using a nested Newton’s method. Let $(\textbf{A}^{(n)},c^{(n)})$ be the $n^{\textrm{th}}$ iteration of the vector of unknowns, where here $\textbf{A}$ is the $({\widetilde{N}}-1)-$dimensional vector of $A_j$ coefficients. Given the *n*th vector of iterates, we find the free surface $\eta (X)=\eta (X;\textbf{A}^{(n)},c^{(n)})$ by satisfying (3.7) at the ${\widetilde{M}}+1$ collocation points

3.12 $$\begin{aligned} X_k=\frac{(k-1)\pi }{{\widetilde{M}}}~~~~\textrm{for}~~~k=1,\ldots ,M+1\,. \end{aligned}$$

Then, substituting this form for $\eta$ along with the velocity potential (3.11) into (3.8) gives the equations

3.13 $$\begin{aligned} 0=F_m(\textbf{A}^{(n)},c^{(n)}):=\int _0^\pi Q(X,\eta (X,\textbf{A}^{(n)},c^{(n)});\textbf{A}^{(n)},c^{(n)})\,\sin (m X)\,\textrm{d}X, \end{aligned}$$

for $m=[1,{\widetilde{M}}]$. These integrals are evaluated using an ${\widetilde{M}}$-point Fourier transform, which gives ${\widetilde{M}}$ independent relations, since $\eta$ is evaluated at the points (3.12). To evaluate the integral accurately, we require ${\widetilde{M}}>2{\widetilde{N}}$.

By considering only the equations for $m\in [1,{\widetilde{N}}-1]$, we then arrive at ${\widetilde{N}}-1$ independent relations for the ${\widetilde{N}}$ unknowns, and so we need one final equation which comes from (3.10) as

3.14 $$\begin{aligned} W(\textbf{A}^{(n)},c^{(n)}):=2\epsilon -\eta (0;\textbf{A}^{(n)},c^{(n)})+\eta (\pi ;\textbf{A}^{(n)},c^{(n)})=0\,. \end{aligned}$$

These ${\widetilde{N}}$ equations are then updated using Newton iterations of the form

$$\begin{aligned} \left( \begin{array}{c} \textbf{A}^{(n+1)} \\ c^{(n+1)}\end{array} \right) =\left( \begin{array}{c} \textbf{A}^{(n)} \\ c^{(n)}\end{array} \right) -\left[ \begin{array}{cc} \dfrac{\partial \textbf{F}}{\partial \textbf{A}} &  \dfrac{\partial W}{\partial \textbf{A}} \\ \dfrac{\partial \textbf{F}}{\partial c} &  \dfrac{\partial W}{\partial c}\end{array} \right] ^{-1} \left( \begin{array}{c} \textbf{A}^{(n)} \\ c^{(n)}\end{array} \right) , \end{aligned}$$

where $\textbf{F}$ is the vector of $F_m$ values for $m\in [1,{\widetilde{N}}-1]$, defined in (3.13), and the entries of the Jacobian *J* are given in Appendix A.

**Fig. 9:**
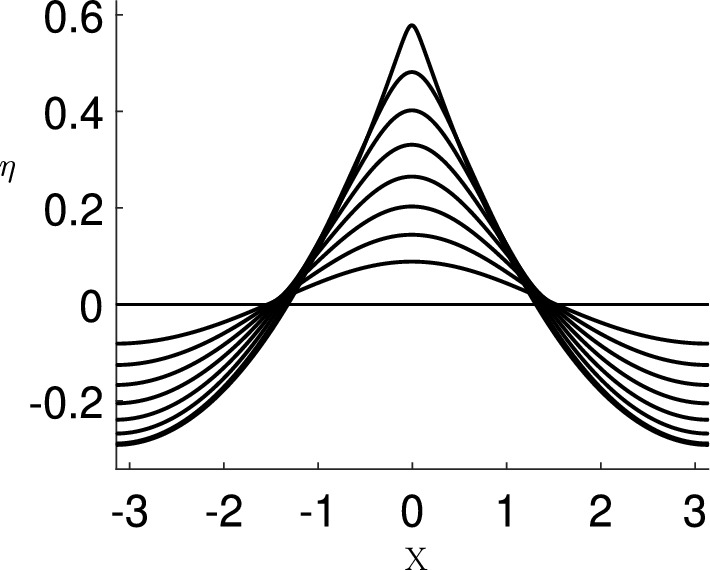
The calculated free surface of the basic state for wave steepness values $\epsilon =0.085$, 0.135, 0.185, 0.235, 0.285, 0.335, 0.385 and 0.435, using the algorithm of Sect. 3.1

Examples of the calculated free surface using the code are shown in Fig. 9, for a sequence of wave steepness values.

### Linear Stability Problem

The linear stability problem is derived by adding a perturbation to the basic state and linearising. For the base flow calculated in Sect. 3.1 to be stationary relative to the moving frame, we need to define a new velocity potential

$$\begin{aligned} \Phi =\phi -cx\,. \end{aligned}$$

We introduce small perturbations to the base flow from Sect. 3.1,

3.15 $$\begin{aligned} \eta (X,t)= &   \overline{\eta }(X)+\eta '(X,t), \end{aligned}$$

3.16 $$\begin{aligned} \Phi (X,z,t)= &   \overline{\Phi }(X,z)+\Phi '(X,z,t), \end{aligned}$$

where an overbar denotes the basic state and the primes denote small perturbations, such that $|\eta '|\ll |\overline{\eta }|$ and $|\Phi '|\ll |\overline{\Phi }|$.

Substituting these expansions into (3.1)–(3.4) and linearising leads to the following equations for the perturbation quantities,

3.17 $$\begin{aligned} \nabla ^2\Phi '= &   0~~~~~z<\overline{\eta }(X), \end{aligned}$$

3.18 $$\begin{aligned} \eta '_t= &   \left( \overline{\Phi }_{zz}-\overline{\eta }_{X}\overline{\Phi }_{Xz}\right) \eta '-\overline{\eta }_{X}\Phi '_{X}-\overline{\Phi }_{X}\eta '_{X}+\Phi '_{z}, \end{aligned}$$

3.19 $$\begin{aligned} \Phi '_t= &   -\overline{\Phi }_{X}\Phi '_{X}-\overline{\Phi }_{z}\Phi '_{z}-\left( 1+\overline{\Phi }_{X}\overline{\Phi }_{Xz}+\overline{\Phi }_{z}\overline{\Phi }_{zz}\right) \eta ', \end{aligned}$$

3.20 $$\begin{aligned} \Phi '\rightarrow &   0~~~~\textrm{as}~~~~z\rightarrow -\infty \,, \end{aligned}$$

with (3.18) and (3.19) evaluated at $z={\overline{\eta }}(X)$. For the far field equation, we have just stipulated that $\Phi '$ tends to zero which is equivalent to the normal velocity tending to zero as $z\rightarrow -\infty$. In fact, it ensures that all perturbation velocity components tend to zero in this limit.

For SH modes of the linear stability problem, we seek Fourier series expansions of (3.17)–(3.20) of the form

3.21 $$\begin{aligned} \eta '= &   e^{-\textrm{i}\sigma t}\sum _{j=-\infty }^{\infty } a_{j}e^{\textrm{i}jX}, \end{aligned}$$

3.22 $$\begin{aligned} \Phi '= &   e^{-\textrm{i}\sigma t}\sum _{j=-\infty }^{\infty } b_{j}e^{\textrm{i}jX}e^{|j|z}\,. \end{aligned}$$

The forms (3.21) and (3.22) automatically satisfy (3.17) and (3.20) and so we need to determine $a_{j}$ and $b_{j}$ such that the two free-surface equations are satisfied. Truncating the infinite summation to the finite sum $j\in [-\widehat{M},\widehat{M}]$ and substituting into the free-surface equations results in a generalised eigenvalue problem of the form

3.23 $$\begin{aligned} \textbf{A}\varvec{\zeta }=\sigma \textbf{B}\varvec{\zeta }, \end{aligned}$$

where $\varvec{\zeta }=(a_{j},b_{j})^T$. The matrices $\textbf{A}$ and $\textbf{B}$ are of size $(2\widehat{M}+1)\times (2\widehat{M}+1)$, and we solve this eigenvalue problem by utilising the Galerkin method first laid out by Zhang and Melville [57]. To do this, we take Fourier transforms of (3.18) and (3.19) and then approximate the integrals on the grid

3.24 $$\begin{aligned} X_u= &   \frac{2\pi u}{\nu }~~~~u=0,\ldots ,\nu -1\,. \end{aligned}$$

Doing this, and exchanging the *j* summation with the *u* summation, leads to the following system of equations:

3.25 $$\begin{aligned}  &   \sum _{j=-\widehat{M}}^{\widehat{M}} F_{j-s}\left[ R_{j}^{(1)}\right] a_{j}+\sum _{j=-\widehat{M}}^{\widehat{M}} F_{j-s}\left[ S_{j}^{(1)}\right] b_{j}=\sigma \sum _{j=-\widehat{M}}^{\widehat{M}} F_{j-s}\left[ T_{j}^{(1)}\right] a_{j},\qquad \end{aligned}$$

3.26 $$\begin{aligned}  &   \sum _{j=-\widehat{M}}^{\widehat{M}} F_{j-s}\left[ R_{j}^{(2)}\right] a_{j}+\sum _{j=-\widehat{M}}^{\widehat{M}} F_{j-s}\left[ S_{j}^{(2)}\right] b_{j}=\sigma \sum _{j=-\widehat{M}}^{\widehat{M}} F_{j-s}\left[ T_{j}^{(2)}\right] b_{j},\qquad \end{aligned}$$

where

3.27 $$\begin{aligned} R_{j}^{(1)}= &   -\overline{\Phi }_{zz}+\textrm{i}j\overline{\Phi }_{X}+\overline{\eta }_{X}\overline{\Phi }_{Xz}, \end{aligned}$$

3.28 $$\begin{aligned} S_{j}^{(1)}= &   \left( \textrm{i}j\overline{\eta }_{X}-|j|\right) e^{|j|\overline{\eta }}, \end{aligned}$$

3.29 $$\begin{aligned} T_{j}^{(1)}= &   \textrm{i}, \end{aligned}$$

3.30 $$\begin{aligned} R_{j}^{(2)}= &   1+\overline{\Phi }_{X}\overline{\Phi }_{Xz}+\overline{\Phi }_{z}\overline{\Phi }_{zz}, \end{aligned}$$

3.31 $$\begin{aligned} S_{j}^{(2)}= &   \left( \textrm{i}j\overline{\Phi }_{X}+|j|\overline{\Phi }_{z}\right) e^{|j|\overline{\eta }}, \end{aligned}$$

3.32 $$\begin{aligned} T_{j}^{(2)}= &   \textrm{i}e^{|j|\overline{\eta }}, \end{aligned}$$

and

3.33 $$\begin{aligned} F_{j-s}\left[ f_{j}\right] =\sum _{u=0}^{\nu -1} f_{j}e^{\textrm{i}(j-s)X_u}\,. \end{aligned}$$

To compute the eigenvalues $\sigma$ and eigenvectors $\varvec{\zeta }$ in (3.23), we use the MATLAB |eigs| command. The value of $\widehat{M}$ is increased until the eigenvalues converge, and we are restricted to $\widehat{M}<\nu /2$. Here, $\nu$ is a parameter which controls the accuracy of the discrete Fourier Transform above, and we increase this value until the $\widehat{M}$ Fourier coefficients we require have converged.

**Fig. 10:**
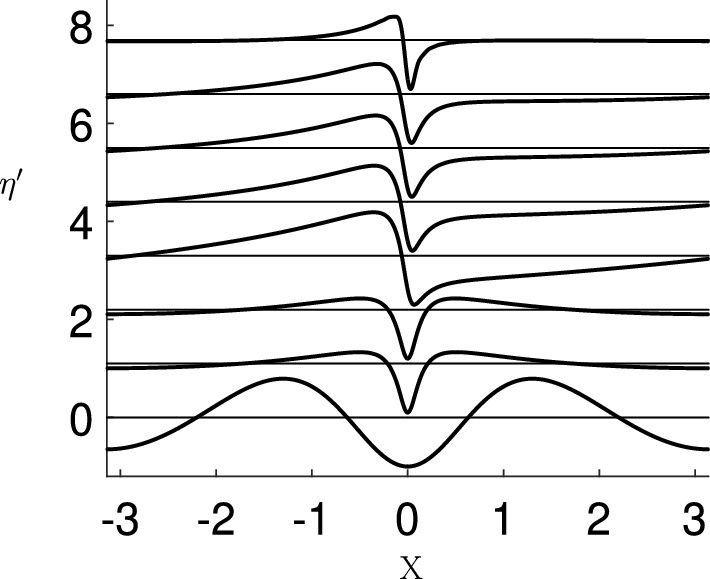
The free surface $\eta '$, associated with the lowest frequency eigenfunction and eigenvalue computed from (3.23), plotted at wave steepness values $\epsilon =0.2$, 0.4285, 0.4290, 0.4295, 0.4300, 0.4305, 0.4310, and 0.4377. Each eigenfunction is normalised so that $\max _X(|\eta '|)=1$

Example computations which show the change in shape of the eigenfunction as $\sigma$ goes from stable to unstable are shown in Fig. 10. In this figure, the eigenfunction free surface $\eta '$ is plotted at a sequence of wave steepness values, given in the caption. The eigenfunctions have been normalised such that $\max _X(|\eta '|)=1$ and each plot has been separated by a constant so they can be all included in the same plot. The wave steepness of the basic state increases going upwards in the figure.

The bottom plot in Fig. 10 is the lowest frequency eigenfunction of the system when the basic state is at low amplitude and the flow clearly is SH stable. Following the progression of this eigenfunction as the wave steepness increases we see the localisation of the eigenfunction near the crest, and in the top plot, which is close to the steepest wave, we see the pronounced dipole structure in the eigenfunction. The stability threshold occurs at $\epsilon \approx 0.4292$, and this is where the structure of the eigenfunction undergoes a qualitative change and corresponds to the maximum of the wave energy as depicted in Fig. 3. Longuet-Higgins and Tanaka [41] show that as the wave steepness is increased, other higher frequency eigenfunctions can also transition into unstable SH modes, and show these modes too have a dipole structure.

## Direct Numerical Simulation via Basilisk

In this section, we begin to test the emergence of wave breaking to the presence of real-world perturbations. For the direct numerical simulation, we use the Basilisk software package [26, 46, 47]. It is the incompressible two-phase version, with the upper fluid set to be air and the lower fluid set to be water. The use of potential solutions with different formulations and approximations as initial conditions for the two-phase flow problem has shown to provide a good description of the basic properties of wave breaking such as the overturning of the water jet, splash-up and gas entrainment, induced vortex-like motion around surface and energy dissipation, examples are Chen et al. [9], Iafrati [28], Iafrati [29], Deike et al. [15], Deike et al. [16] and Mostert at al. [43].

The governing equations are the Navier–Stokes equations with surface tension, written here in dimensional form,

4.1 $$\begin{aligned} \rho \left( \frac{\partial {\textbf{u}}}{\partial t} + {\textbf{u}} \cdot \nabla {\textbf{u}}\right) = -\nabla p + \mu \nabla \cdot \left( \nabla {\textbf{u}} + (\nabla {\textbf{u}})^T \right) + \rho g {\textbf{e}}_3+ {\gamma } \kappa \delta _s \mathbf {{\widetilde{N}}}\,, \end{aligned}$$

together with the conservation of mass

4.2 $$\begin{aligned} \nabla \cdot {\textbf{u}} = 0\,, \end{aligned}$$

with density a Lagrangian invariant

4.3 $$\begin{aligned} \frac{\partial \rho }{\partial t} + \textbf{u}\cdot \nabla \rho = 0\,, \end{aligned}$$

where ${{\textbf {u}}}$ is the fluid velocity, $\rho$ the fluid density, $\mu$ the dynamic viscosity, *p* the pressure, *g* the acceleration due to gravity, and ${\textbf{e}}_3$ the unit vector in the vertical $z-$direction, ${\gamma }$ denotes the surface tension coefficient, $\kappa$ represents the curvature of the interface, and $\mathbf {{\widetilde{N}}}$ the unit normal vector to the interface. The Dirac delta function $\delta _s$ captures the localization of the surface tension term at the fluid interface. The boundary conditions at the interface are the vanishing of the tangential and normal stresses, with the latter balanced by the surface tension force (cf. Scardovelli and Zaleski [51]). As in the previous sections, the coordinate system is oriented so that *z* points vertically, and *x* is the horizontal coordinate.

The Bell–Colella–Glaz scheme [3] is used for the nonlinear advection term, and the diffusion is treated implicitly. The volume of fluid (VOF) method is used to describe the interface between air and water using a momentum-conserving implementation [48]. The surface fraction $f({\textbf{x}}, t)$ indicates the fraction of a cell containing water and air. If there is only water, $f = 1$. If there is only air, $f = 0$. Intermediate values $0<f<1$ denote a mixture. The surface fraction *f* is a Lagrangian invariant and so satisfies an equation similar to (4.3).

The direct numerical simulations are initiated with initial data that approximate a SH unstable periodic travelling Stokes wave (the precise form of the initial data is given below). In principle, we would add an eigenfunction from §3, with small amplitude, to the basic state and then set it in motion. However, this is not necessary as the Stokes wave is not an exact solution of the Navier–Stokes equations, so implicit perturbations are added automatically. As long as there is a component of the unstable eigenfunction in the implicit perturbation the observed effects are the same, as this eigenfunction is the most unstable mode and hence will grow fastest, thus becoming the largest correction to the basic Stokes wave. A key part of this paper is to introduce an SVD-based algorithm precisely for this filtering. The difference between the basic Stokes wave and the computed solution is filtered, using a singular value decomposition (SVD), and we look for components that have the dipole structure seen in breaking waves. This dipole structure then turns out to be an approximation to the dipole structure in the exact eigenfunction (see Fig. 10).

The real-world phenomena present in the perturbations are:

- presence of dissipation,
- presence of surface tension,
- approximation error in the basic Stokes wave,
- depth is finite but very large, an approximation to infinite depth,
- inclusion of a non-zero density for the upper fluid,
- fluctuations in the speed of the Stokes wave and perturbation.

### Initial Conditions

As mentioned in the introduction, the choice of the 5th-order solution for our two-phase simulations, instead of the fully numerical solution computed herein, was motivated by balancing the proximity to real-world events offered by low-order analytical theories with the inclusion of higher order harmonics provided by the 5th-order solution, which, as will be shown, are necessary for producing sufficiently steep non-breaking waves.

A fifth-order asymptotic Stokes wave solution due to Fenton [22] for the surface profile and velocity potential is used as initial condition for our simulations. The analytical formula computed by Fenton, for the case of inviscid, irrotational, periodic waves propagating without change of form over a fluid layer of depth $h_0$, is given by

4.4 $$\begin{aligned} \begin{aligned} k \eta (x) =&\; k h_0 + \epsilon \cos (k x) + \epsilon ^2 B_{22} \cos (2 k x) + \epsilon ^3 B_{31} (\cos (k x) - \cos (3 k x)) \\&+ \epsilon ^4 \left( B_{42} \cos (2 k x) + B_{44} \cos (4 k x)\right) \\&+ \epsilon ^5 \big (-(B_{53} + B_{55}) \cos (k x) + B_{53} \cos (3 k x) + B_{55} \cos (5 k x)\big ) + \mathcal {O}(\epsilon ^6). \end{aligned} \end{aligned}$$

The dimensionless velocity potential of the travelling wave in a fixed frame of reference is

4.5 $$\begin{aligned} \phi (x,z,t) = \sqrt{\frac{g}{k^3}} C_0 \sum _{i=1}^{{5}} \sum _{j=1}^{i} \epsilon ^{i} A_{ij} \cosh (j k z) \sin (j k x) + \mathcal {O}(\epsilon ^6). \end{aligned}$$

Here, $x$ and $z$ denote the horizontal and vertical coordinates, respectively. The parameter $\epsilon = kH/2$, with $k$ being the wavenumber and *H* the trough-to-crest wave height, is the non-dimensional steepness parameter from (3.10) and is the small expansion parameter. $C_0 = \tanh (kh_0)$ is the non-dimensional linear wave speed. The coefficients $A_{ij}$ and $B_{ij}$, which are functions of $kh_0$ only, are given in Fenton [22].

The use of the following analytical expression enables a straightforward and compact initialisation of the air–water interface location and the velocity components in water. Additionally, it avoids the uncertainties associated with adapting the numerical solution to fit the required input format for the DNS and estimating the velocity components using the numerical method described in §3.1.

### Numerical Configuration

The wave propagates from left to right along the *x*-axis, in a square box of length $L = \lambda = 1$, where $\lambda$ denotes the wavelength, allowing us to impose periodic boundary conditions in the direction of propagation, along with free slip conditions at the top and bottom walls. The simulation is conducted using non-dimensional parameters: $\epsilon = kH/2$, half the nondimensional crest-to-trough steepness parameter and small expansion parameter; $\rho _a/\rho _w = 1/850$, the density ratio between air and water; $\mu _a/\mu _w = 17.4 \times 10^{-6} / 8.9 \times 10^{-4}$, the viscosity ratio; Re = $c\lambda /{\nu _w}$, the Reynolds number in water, with $\nu _w$ is the kinematic viscosity of water, $c = \sqrt{g/k}$ is the deep-water linear phase speed, and $k=2\pi /\lambda =2\pi$ the wavenumber; and $\textrm{Bo}=\Delta \rho g/{\gamma } k^2$ with $\Delta \rho = \rho _w - \rho _a$, the Bond number. The water depth is set to $h_0/\lambda = 1/2$, which is just at the threshold of deep-water conditions.

A fixed 2D Cartesian mesh is used where the number of cells per dimension is expressed as $N = 2^l$. For the simulations presented in this work, we set $l$ to values $l = 9$, $l = 10$, and $l = 11$ to verify convergence.

In this configuration, two definitions of the physical length scale of the Stokes wave can be established based on the Reynolds number, which characterises the balance between viscous and inertial forces, and the Bond number, which defines the balance between surface tension and gravitational forces. This study will explore how these two dimensionless parameters influence the onset of wave breaking.

The total number of simulations conducted is summarised in Table 1.

**Table 1:** Summary of simulation runs

Set	$\epsilon$	Re	Surface tension (Bo)	Order	Refinement level
S1	0.3–0.39	$4\times 10^4$	Off $(\textrm{Bo}\rightarrow \infty )$	1–5	9, 10, 11
S2	0.15, 0.25, 0.42	$4\times 10^4$	Off $(\textrm{Bo}\rightarrow \infty )$	5	11
S3	0.39	$1, 2, 4, 8\times 10^4$	Off $(\textrm{Bo}\rightarrow \infty )$	5	11
S4	$0.39$	$4\times 10^4$	$100, 500, 1000, 2000$	5	$11$

The set S1 consists of $$3\times 50$$ simulations, each varying the initial steepness incrementally by $$\Delta \epsilon = 0.01$$ across the range [0.3, 0.39]. These variations were applied to each order of approximation of the initial solution (4.4)–(4.5), and for each refinement level. Results concerning the breaking threshold will be presented in Sect. 5.2. The set S2, which consists of 3 simulations, was used together with simulations from S1 to assess the role played by the initial steepness on the development of the instability; it will be discussed in Sect. 5.3. The set S3, which includes $$4$$ simulations, is focussed on exploring the effects of viscosity in Sect. 5.5. Here, the Reynolds number was set to values of $$10^{4} $$, $$2\times 10^{4}$$, $$4\times 10^{4}$$ and $$8\times 10^{4}$$. The set S4 consists of simulations conducted to investigate the effects of surface tension on the development of the instability by varying the Bond number, $$\textrm{Bo}$$, across the values $$100$$, $$500$$, $$1000$$, and $$2000$$. Here small surface tension corresponds to high Bond number and vice versa. In all simulations from the previous sets, the surface tension contribution was disabled in the two-phase Navier–Stokes solver to isolate other factors, while results from S4 will be qualitatively compared with the case where surface tension is absent.

### Instability Quantification

We quantify the evolution of instabilities by defining the variable $I(x,t)$

4.6 $$\begin{aligned} I(x,t) = \eta _{\text {stat}}(x,t) - \eta (x,0), \end{aligned}$$

where $\eta _{\text {stat}}(x,t) = \eta (x - U(t)t, t)$ is an approximation to the stationary wave, and represents the wave profile translated by the instantaneous crest velocity $U(t)$. This velocity is determined by fitting a third-order polynomial to the crest’s location over time and then differentiating it to obtain the velocity.

While more sophisticated methods exist for instantaneous analysis in space and time, such as those employing the Hilbert transform or the wavelet transform to estimate phase velocity, our approach has yielded a relatively satisfying stationary wave propagation for the purposes of identifying the unstable eigenfunction.

## Results of the Simulations

In this section, we look at the correlation between wave breaking and key input parameters. We classify a wave as breaking if the free surface develops a local vertical interface within the first three linear time periods $[0,3T_0]$, where $T_0= 2\pi /\sqrt{gk}$. Extensive testing has confirmed that extending this observation period up to the first seven time periods, $7T_0$, does not alter the classification of a wave from non-breaking to breaking.

### Effect of the Grid Resolution

We initially examine the effect of numerical resolution by employing different numbers of grid points per wavelength: $N=2^9$, $N=2^{10}$, and $N=2^{11}$. A proper simulation of surface waves requires a fine space resolution, particularly at the interface and within the boundary layer at the bottom of the domain where energy dissipation occurs. In their study, Deike et al. [15] achieved numerical convergence in terms of total energy integrated over the entire domain with a Reynolds number of Re $=4\times 10^4$ using $l=9$.

However, as noted by Iafrati et al. [30], wave breaking does not consistently occur under the same initial conditions when different resolutions are employed. This inconsistency was confirmed in our work, where the investigated numerical resolutions showed that wave breaking is either not detected or delayed when the resolution is doubled. This delay is likely due to improved resolution of the initial higher order harmonics. Consequently, we have chosen to focus on runs with the highest resolution, $N=2^{11}$, for a more accurate analysis.

### Effect of the Approximation Order of the Initial Condition

We then examine the effect of the order of approximation of the initial conditions (4.4) and (4.5) on wave breaking. For each order of approximation, as defined by the power of the expansion parameter $\epsilon$ in (4.4) for the free-surface elevation and the value of *i* in (4.5) for the velocity potential expressed as a series expansion, the series are truncated to the corresponding order and then input into the Navier–Stokes solver. The results, shown in Table 2, indicate that breaking is first detected at a steepness value of $\epsilon = 0.33$ for the linear wave (first order). The threshold increases with the order of approximation: $\epsilon =0.34$ for the second order, $\epsilon =0.36$ for the third, $\epsilon = 0.38$ for the fourth, and finally $\epsilon = 0.39$ for the fifth-order approximation. This pattern confirms that higher order harmonics, despite their small amplitudes in the initial solution, enable the formation of steeper non-breaking waves. It suggests that using higher order solutions, such as the fully nonlinear one computed in Sect. 3.1, might allow waves to approach the theoretical steepness limit of $\epsilon \approx 0.43$ where the linear theory predicts breaking should occur, as discussed in Sect. 3.2.

**Table 2:** Breaking threshold after $3T_0$ using $l=11$. $(\circ )$ represents a non-breaking event, while (✕) represents a breaking event

$\epsilon$	*E*	O1	O2	O3	O4	O5
0.30	0.0431	$\circ$	$\circ$	$\circ$	$\circ$	$\circ$
0.31	0.0458	$\circ$	$\circ$	$\circ$	$\circ$	$\circ$
0.32	0.0486	$\circ$	$\circ$	$\circ$	$\circ$	$\circ$
0.33	0.0514	✕	$\circ$	$\circ$	$\circ$	$\circ$
0.34	0.0542	✕	$\circ$	$\circ$	$\circ$	$\circ$
0.35	0.0569	✕	✕	$\circ$	$\circ$	$\circ$
0.36	0.0597	✕	✕	$\circ$	$\circ$	$\circ$
0.37	0.0624	✕	✕	✕	$\circ$	$\circ$
0.38	0.0651	✕	✕	✕	✕	$\circ$
0.39	0.0676	✕	✕	✕	✕	✕

The value of the energy *E* is taken from Fig. 3

**Fig. 11:**
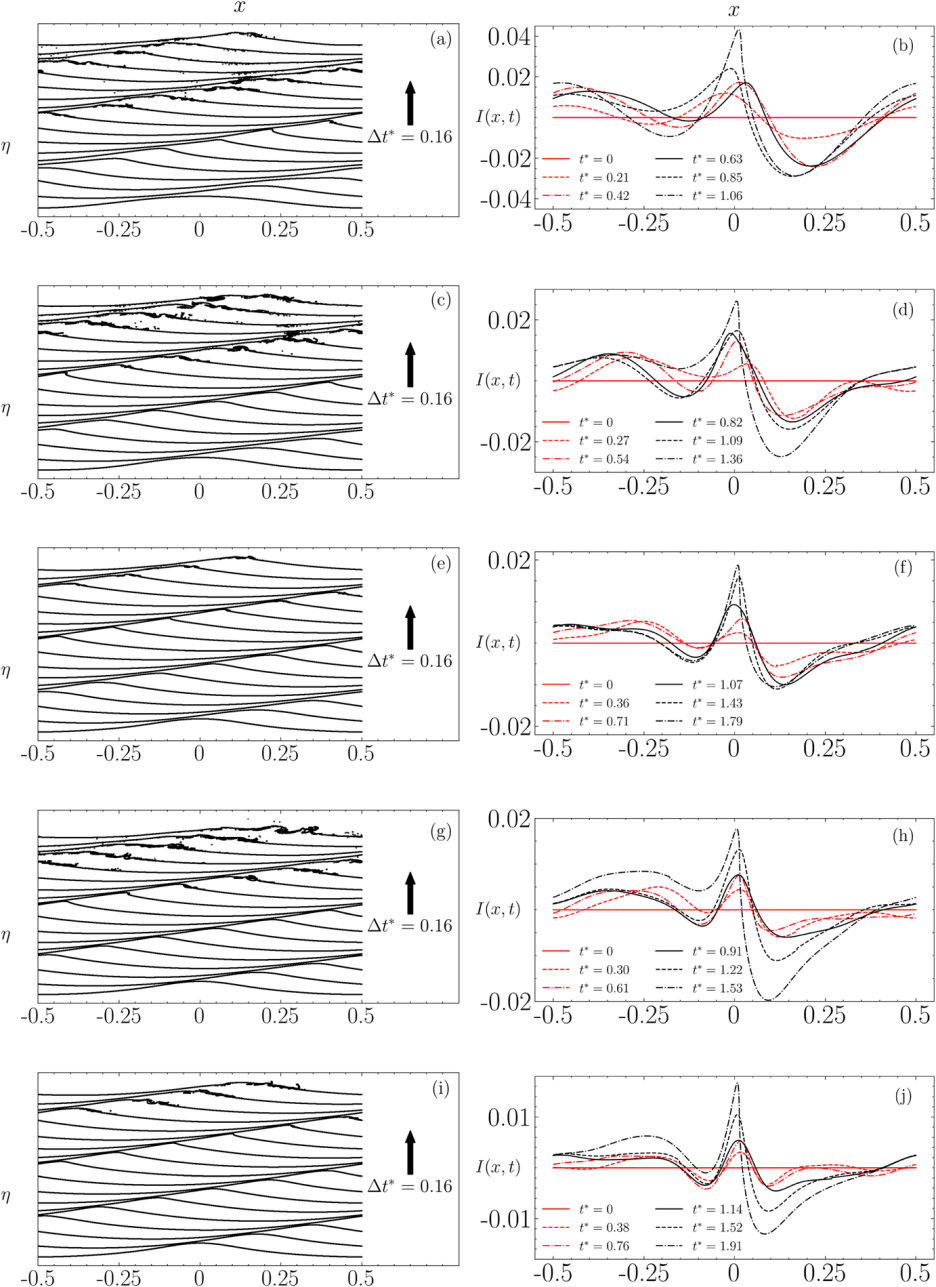
Evolution of the wave profile (left) and the corresponding computed instability $I(x,t)$ (right) at the breaking threshold for each order of approximation to the initial Stokes wave. The results are presented for: **a**, **b** O1 with $\epsilon = 0.33$, **c**, **d** O2 with $\epsilon = 0.35$, **e**, **f** O3 with $\epsilon = 0.37$, **g**, **h** O4 with $\epsilon = 0.38$, and **i**, **j** O5 approximation with $\epsilon = 0.39$

Figure 11 displays the free-surface profiles up to and beyond the wave breaking time for different non-dimensional times $t^* = t/T_0$ with $T_0 = 2\pi /\sqrt{gk}$, alongside the evolution of $I(x,t)$ which is only shown up to a few time steps before wave breaking occurs. Across all orders of approximation, there is a noticeable transformation from an initially nearly symmetric instability concentrated at the wave crest into a dipole-like form similar to that seen in Fig. 10. This transformation features increasing instability at the wave’s rear and a decrease at the front, highlighting the dynamic changes leading up to wave breaking.

**Fig. 12:**
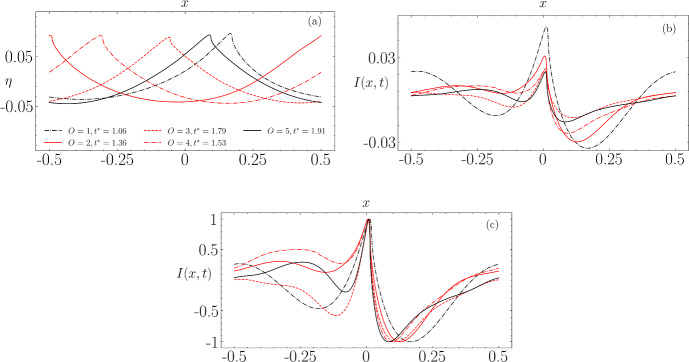
**a** Wave profile and **b** corresponding instability profile at the time of breaking. **c** Instability profile from (**b**) scaled to the range $[-1, 1]$ *for comparison*

In Fig. 12, we present the wave profiles at the point of breaking, along with the computed instabilities. For comparison, these instabilities are scaled to the range $[-1, 1]$. A key observation is the correlation between the order of approximation and both the time $t^*$ required for the instability to reach its maximum amplitude, and the amplitude of the peak value prior to wave breaking. Notably, except for the $3^{\textrm{rd}}$-order solution, which deviates from this pattern, higher accuracy in the solution generally results in a longer duration before the instability achieves its dipole-like form prior to breaking. This trend suggests that more precise initial conditions delay the onset of significant instability, which is not altogether surprising as a more accurate initial wave approximation to the Stokes wave would have a significantly smaller amount of noise added to it, and it is this noise which grows in time, leading to wave breaking.

Figure 12c displays the instability scaled to $[-1, 1]$. The first- and third-order solutions exhibit similar characteristics, specifically a decrease in amplitude at the left boundary, whereas the amplitudes in the other orders of approximation increase in this region. The fifth-order solution’s instability, in comparison, is more localised near the crest and forms the most pronounced dipole shape among the examined solutions. These variations in instability structure, leading up to the dipole formation, can be attributed to different types of nonlinear interactions among the wave components, including three-wave, four-wave, and higher order interactions. Consequently, we have chosen to concentrate on the fifth-order solution to explore how changes in the initial steepness and Reynolds number influence the development of the instability.

### Effect of Initial Steepness

Now that we have characterised the effect of the order of approximation on the development of the instability, for a range of breaking cases, a legitimate question is: How does this compare to the theoretical description of the SH instability phenomenon and its role in triggering wave breaking in the actual configuration?

**Fig. 13:**
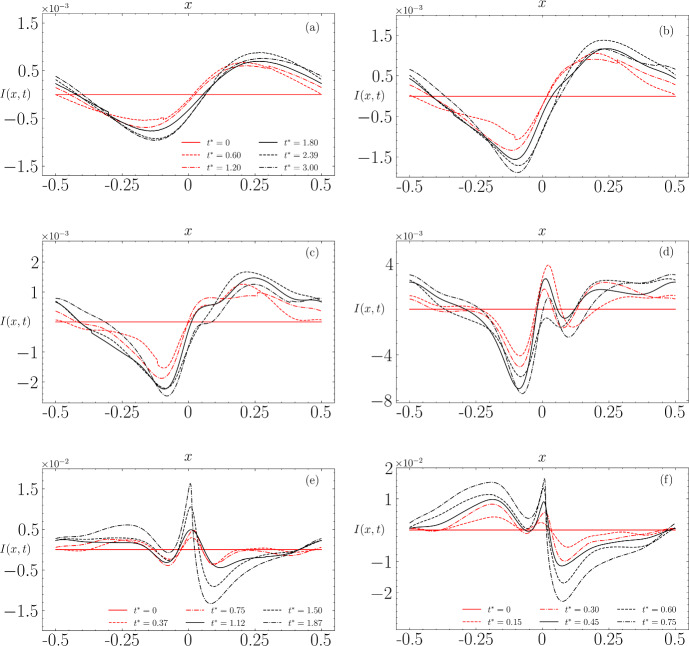
Time evolution of instabilities obtained with the $5^{\textrm{th}}$ order initial condition using different steepness values. In **a** $\epsilon = 0.15$, **b** $\epsilon = 0.25$, **c** $\epsilon = 0.3$, and **d** $\epsilon = 0.37$, the same time steps are shown for non-breaking cases. In **e** $\epsilon = 0.39$ and **f** $\epsilon = 0.42$, the time steps are uniformly distributed between $t = 0$ and the time when breaking occurs

To address this question, we compute the instability signal using the $5^{\textrm{th}}$ order approximation initial condition for a set of initial steepness values. Figure 13 shows the instability signals obtained with the $5^{\textrm{th}}$ order solution using different initial steepness values.

In panel (a), corresponding to $\epsilon = 0.15$ (thus low amplitude), we observe a sudden evolution of the spatial form of the instability from initially null to a signal resembling a sinusoid, with an amplitude relatively small compared to those obtained for higher amplitudes. This is consistent with the wave propagating stably. As the steepness increases to $\epsilon = 0.25$ (panel (b)), the amplitude of the instability is more significant, and the maxima and minima of the sinusoid are more pronounced. For $\epsilon = 0.3$ (panel (c)), the signal still retains its sinusoidal structure but begins to show undulations that may stem from another type of instability. At $\epsilon = 0.37$ (panel (d)), the contributions of the signal containing undulations and the sinusoid are almost equally influential.

At $\epsilon = 0.39$ (panel (e)), which corresponds to the steepness at which the wave breaks, the structure of the instability is strongly marked by a dipolar structure. For $\epsilon = 0.42$ (panel (f)), where the development from the initial wave to breaking is relatively immediate, the dipolar structure is still present, more pronounced but seems modulated by a signal resembling the opposite of what is obtained for lower $\epsilon$ values. This suggests that there might be two types of nonlinearly interacting instabilities, differing in their spatial forms, steepness at which they are dominant but especially in their evolution times and growth rates.

To verify this hypothesis, in the subsequent section, we attempt to decompose the instability signal to identify the shapes of instabilities capturing specific amounts of energy. This analysis will be conducted using a filter based on the SVD.

### Mode Decomposition Using the Singular Value Decomposition (SVD)

SVD is used to split the instability signal into distinct modes, each representing coherent structures that capture a specific amount of energy. This powerful statistical tool is widely used for dimensionality reduction across various applications, including image compression, recommendation systems, and forecasting [32].

In this study, SVD is applied to the snapshot data matrix ${\varvec{I}}$, which is constructed from the vertical coordinates of the instability signal at each spatial point uniformly distributed across the $x-$domain. The rows of ${\varvec{I}}$ represent the evolution of these coordinates at each time step up to $t_f = 3T_0$ if no vertical interface was detected, or up to $t_f = t_{BR}$ if the wave breaks earlier:

$$\begin{aligned} {\varvec{I}} = \begin{bmatrix} I_{1,1} &  I_{1,2} &  \dots &  I_{1,{M_t}} \\ I_{2,1} &  I_{2,2} &  \dots &  I_{2,{M_t}} \\ \vdots &  \vdots &  \ddots &  \vdots \\ I_{{N_x},1} &  I_{{N_x},2} &  \dots &  I_{{N_x}, {M_t}} \end{bmatrix}. \end{aligned}$$

Here, $N_x$ is the number of spatial points distributed across the *x*-domain, and $M_t$ is the total number of time steps recorded during the simulation. The SVD factorises ${\varvec{I}}$ into three matrices:

$$\begin{aligned} {\varvec{I}} = {\textbf{U}} \cdot \varvec{{S}} \cdot {\textbf{V}}^T, \end{aligned}$$

where ${\textbf{U}}$ consists of the left singular vectors representing the spatial modes, $\varvec{{S}}$ is a diagonal matrix containing the singular values that represent the captured variance of each mode, and ${\textbf{V}}^T$ is the transpose of the right singular vectors representing the temporal evolution of these modes. Both ${\textbf{U}}$ and ${\textbf{V}}$ are unitary matrices comprised of orthogonal space and time singular vectors, respectively [20].

The instability vertical coordinate at spatial point $x_i$ and time $t_j$ is represented as $I_{i,j}$. This can be expressed as

**Fig. 14:**
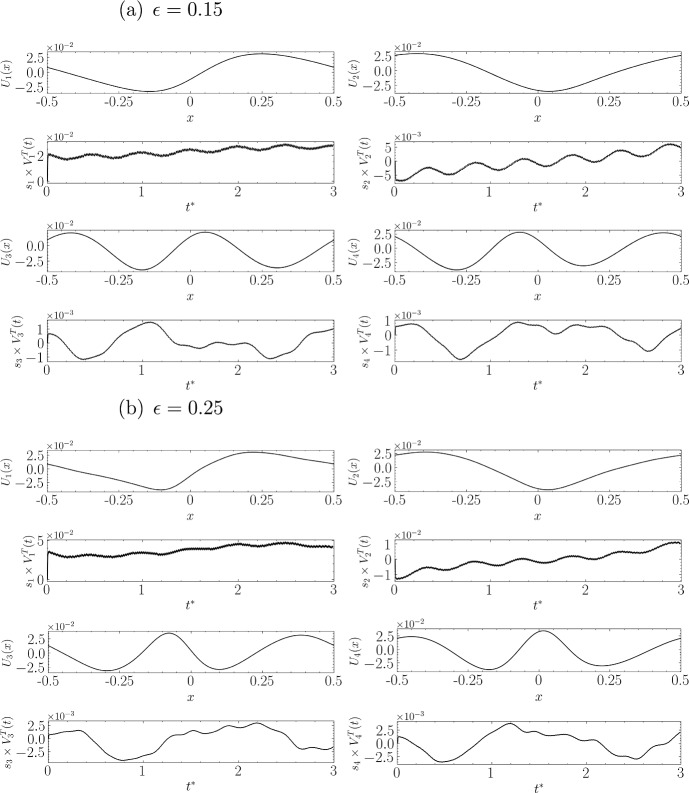
Visual representation of the spatial structures of the first four SVD modes and the temporal evolution of their corresponding amplitudes for two steepness values: **a** $\epsilon = 0.15$ and **b** $\epsilon = 0.25$

**Fig. 15:**
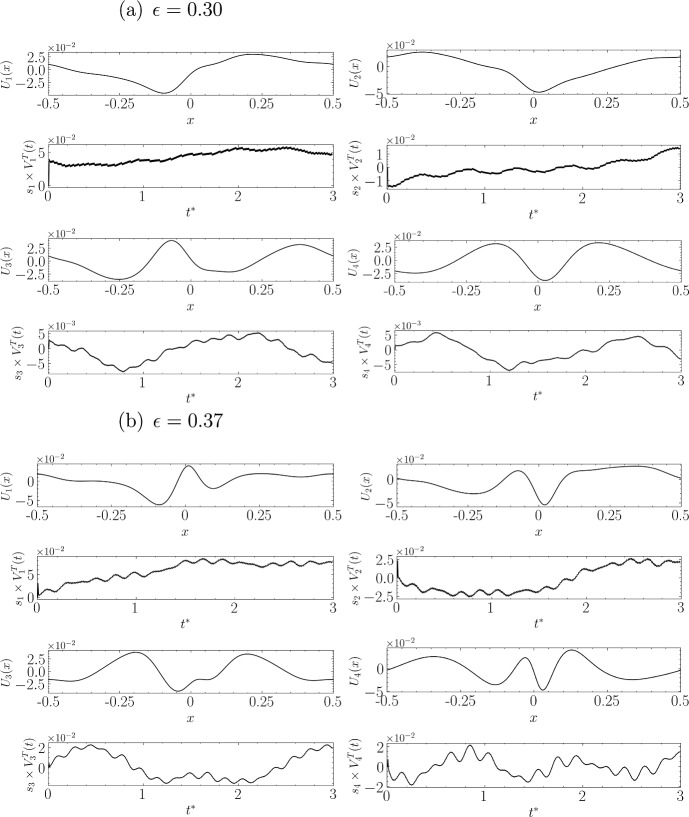
Same as Fig. 14, but for steepness values: **a** $\epsilon = 0.30$ and **b** $\epsilon = 0.37$

**Fig. 16:**
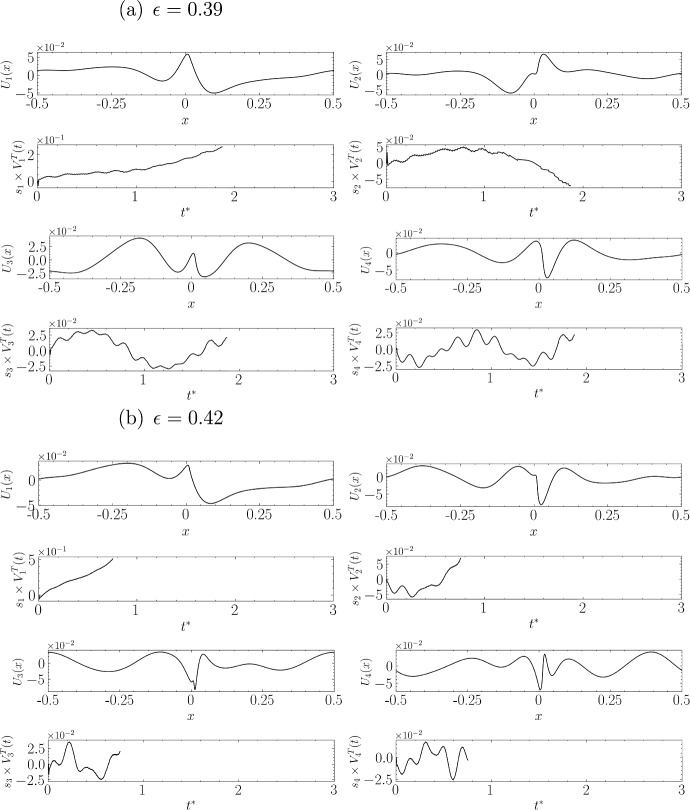
Same as Fig. 14, but for steepness values: **a** $\epsilon = 0.39$ and **b** $\epsilon = 0.42$

5.1 $$\begin{aligned} I_{i,j}=I(x_i,t_j) = \sum _{k=1}^{N_{\text {Components}}} {s}_k U_k(x_i) V_k^{T}(t_j), \end{aligned}$$

where $N_{\text {Components}} = 4$ limits the number of modes retained in this analysis. The SVD was performed using the numpy.linalg.svd function from Python’s NumPy library. Figs. 14, 15, and 16 show the shape (left singular vector) and amplitude evolution (singular value multiplied by the right singular vector) corresponding to the instability for each steepness studied in the preceding section.

In Fig. 14, panel (a) displays the shapes of the first four components and the temporal evolution of their amplitudes. As expected, the dominant sinusoidal shape appears first, exhibiting the largest amplitude compared to the subsequent components. The second component, which is an order of magnitude smaller, appears as a phase-shifted version of the first component. This behaviour is typical of SVD when applied to sinusoidal signals, considering an orthonormal basis of cosine and sine for the corresponding frequencies.

For example, applying SVD to the coordinates of the free surface rather than those of the instability signal, based on the results of the fifth-order solution simulation, assigns to each mode an energy close to the amplitude corresponding to the frequency and wavenumber of each harmonic of the initial solution in two pairs (cosine and sine of each spatial and temporal frequency). For this reason, we now compare the amplitudes of the third and fourth components with that of the first and second.

The next two components, while about 30 times smaller in amplitude than the first, are close to each other and are phase-shifted by $\pi /2$, thus indicating the second pattern that captures energy in the instability. In Fig. 14b for $\epsilon =0.25$, the amplitude is relatively low, even though nonlinear effects begin to be more significant. Despite this, SVD still manages to separate the two distinct energy patterns (the first two are about 20 times more significant than the following two in terms of amplitude).

In Fig. 15a for $\epsilon =0.3$, the nonlinear effects continue to be significant, and we begin to see deformations in the shapes of the components, likely due to interactions with higher frequency modes. This can be easily noticed in the graph showing amplitude variations (frequencies of the fifth harmonic can be recognised, for example). At this stage, the last two components are only 5 times less significant than the first two in terms of amplitude.

Figure 15b for $\epsilon =0.37$ shows very strong nonlinearities, and the shape of the first component, while still retaining some traces of the mode seen at lower amplitudes, now has a new form of instability dominating its structure. This can be interpreted as a kind of merging of the first and third modes, distinct at lower amplitudes. Indeed, the multiplication factor between the amplitudes of the third component and that of the first component has reduced to about 3.5. The same observation also applies if we compare the second and fourth modes at this amplitude with those obtained at lower amplitudes. Obviously, the term “merging” does not precisely represent the potentially complex interactions between different components; simply observing the high frequencies visible in all amplitude graphs gives an idea of the complexity of the nonlinear interactions present here.

Figure 16a for $\epsilon =0.39$ corresponds to the onset of wave breaking, marking the transition from a relatively stable state to an extremely energetic chaotic state causing turbulence. The first mode concentrates all the energy (about 10 times more energy than the last two) and clearly has a dipolar structure.

**Fig. 17:**
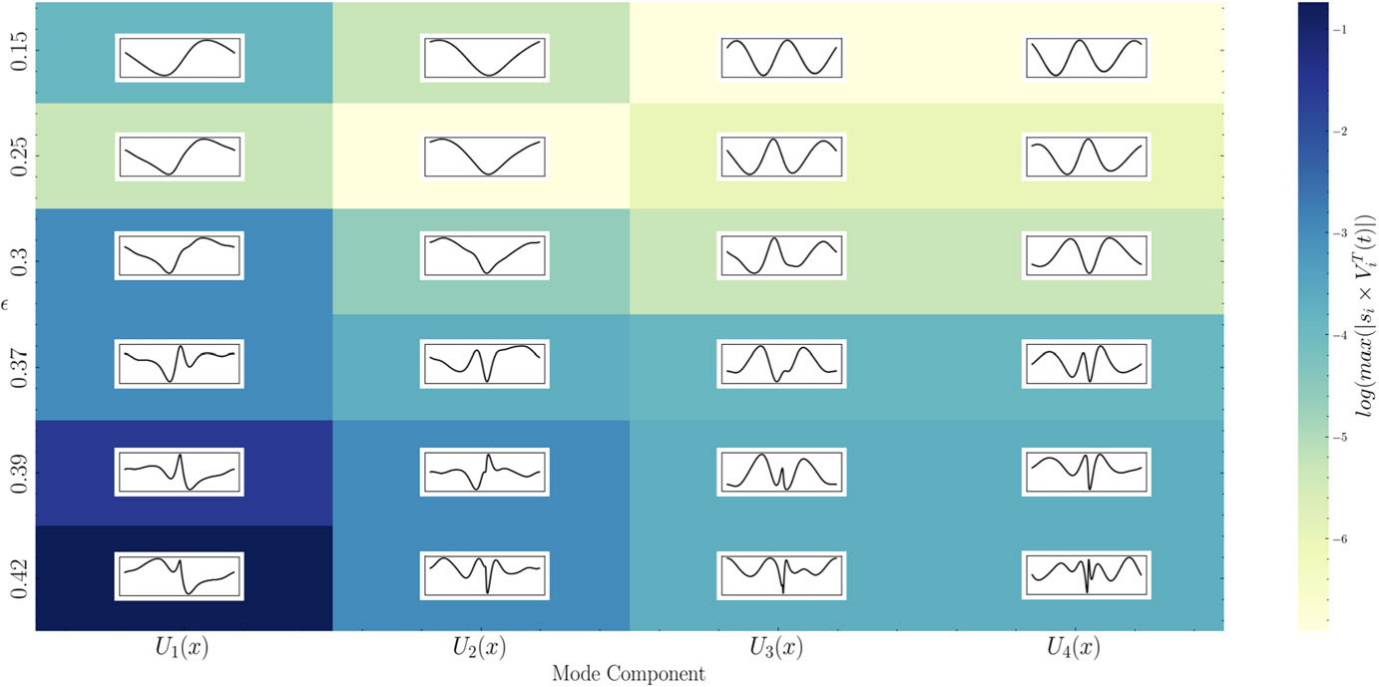
Graph showing the 4 principal components of instabilities for each steepness value ordered by amplitude in $\log$ scale

In Fig. 16b for $\epsilon =0.42$, the first component reaches an amplitude about 20 times greater than the last two, it has a predominantly dipolar structure, but this structure seems to be modulated by a signal that closely resembles the dominant mode at low amplitude reversed in sign. The different components are gathered in Fig. 17 allowing a visual representation of the potential interaction occurring when the amplitude (equivalently energy) of each of them varies.

The fact that two (or more) competing instabilities are present in Stokes waves is fairly common and has been studied by several authors. These are generally classified as either Class I or Class II. In our case, it is complicated to precisely characterise the two instabilities and the interaction modes responsible for them. First, this complexity arises because we stabilise the wave by estimating the crest speed, which in a way biases the wave phase. Furthermore, even though the SVD effectively distinguishes the modes at low amplitudes, the constraint of orthogonality in the mathematical sense may not hold much physical significance when nonlinearities are strong. This could be another source of uncertainty when the modes have energies that are quite close to each other.

**Fig. 18:**
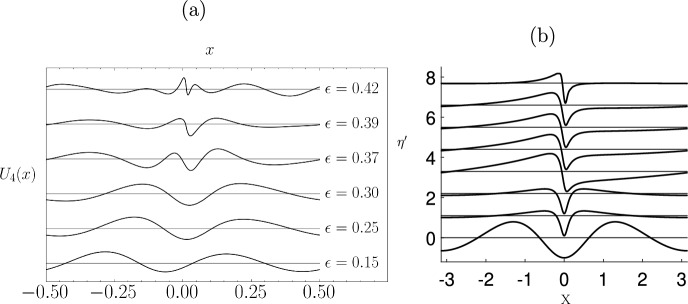
Comparison between the linear stability from Sect. 3.2 and results and the SVD $4^\textrm{th}$ mode $U_4(x)$

Figure 18 serves as the central illustration for this section, wherein we compare the shapes of SH modes obtained through linear instability analysis discussed in Sect. 3.2 with the shapes of the fourth mode (third in terms of energy) derived via SVD applied to our instability signal. Additionally, each mode is constrained such that $\max ( |U(x)|)=1$.

As previously explained, when nonlinearities become significant especially near the wave-breaking threshold, the modes begin to merge under the constraint of orthogonality. Figure 18 tells us that even when considering only the mode capturing the third-highest energy level (with the maximum being captured by the dipole at $\epsilon = 0.39$ and $\epsilon = 0.42$), we can reproduce a scenario that closely approximates the theoretical description of SH instability. This suggests that it is indeed one of the mechanisms contributing effectively to wave breaking.

### Effect of Viscosity

As stated in Sect. 4.2, the water viscosity parameter influences two key non-dimensional parameters: the viscosity ratio $\mu _a/\mu _{w}$ and the Reynolds number in water, $\textrm{Re}$. Since our focus is on water waves, the viscosity ratio $\mu _a/\mu _w$ is kept constant, allowing us to isolate the effects of viscosity by varying the Reynolds number, defined as $\textrm{Re} = c \lambda /\nu _w$. In the absence of surface tension, the characteristic length scale of the flow is taken as the wavelength $\lambda$. Varying the Reynolds number modifies the balance between viscous and inertial forces in the flow. The values taken for the Reynolds number are Re $=1\times 10^4$, Re $=2\times 10^4$, Re $=4\times 10^4$ and Re $=8\times 10^4$, corresponding to dimensional wavelength of $\lambda \sim 2.25~\textrm{cm}$, $\lambda \sim 4.5~\textrm{cm}$, $\lambda \sim 9~\textrm{cm}$ and $\lambda \sim 18~\textrm{cm}$.

**Fig. 19:**
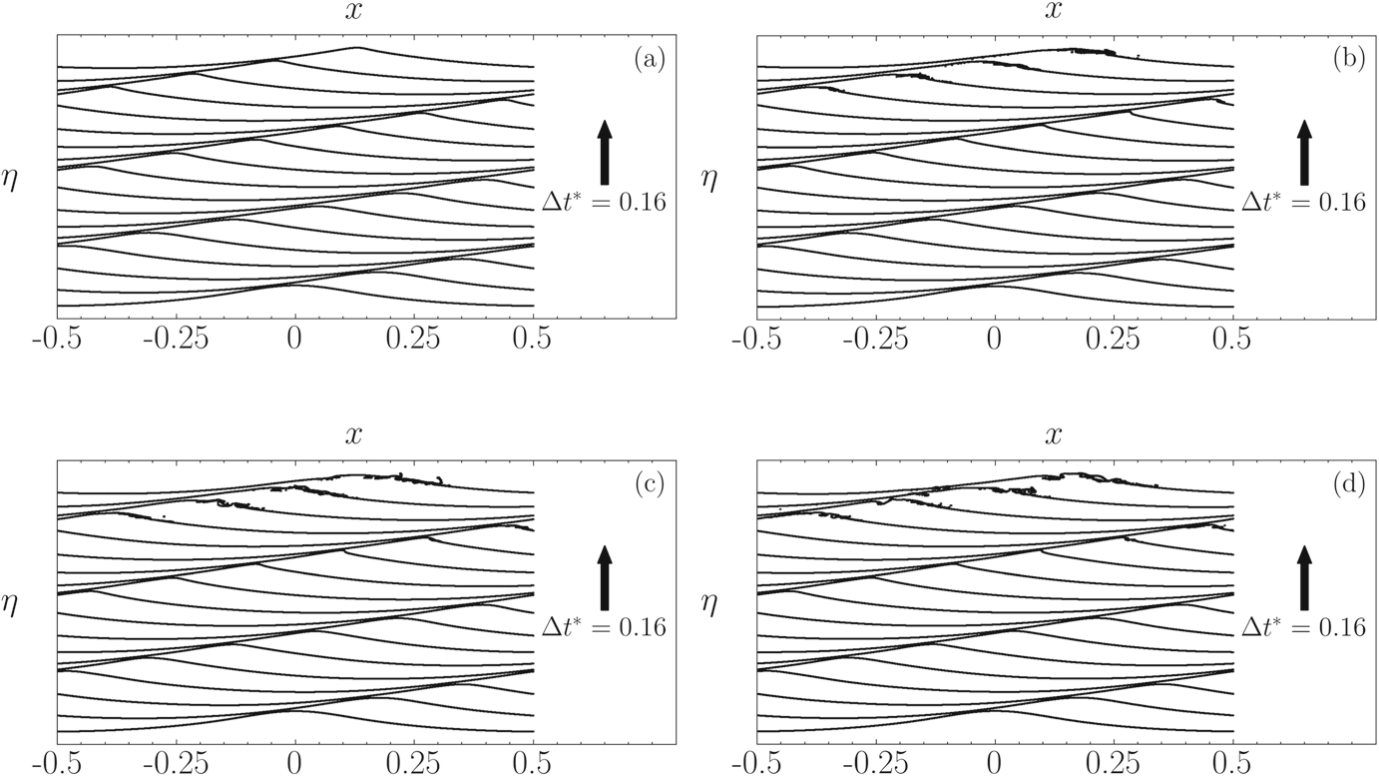
Evolution of free-surface profiles using different Reynolds number values: **a** Re $=$ $1\times 10^4$, **b** Re $=$ $2\times 10^4$, **c** Re $=$ $4\times 10^4$, **d** Re $=$ $8\times 10^4$

Figure 19 illustrates the evolution of free surface profiles obtained using a fifth-order initial solution initialised with $\epsilon =0.39$ and varying Reynolds numbers. Notably, the case initialised with Re $=1\times 10^4$, which has the highest viscosity, does not evolve into wave breaking. In contrast, cases with higher Reynolds numbers, indicating lower viscosity, do lead to wave breaking.

**Fig. 20:**
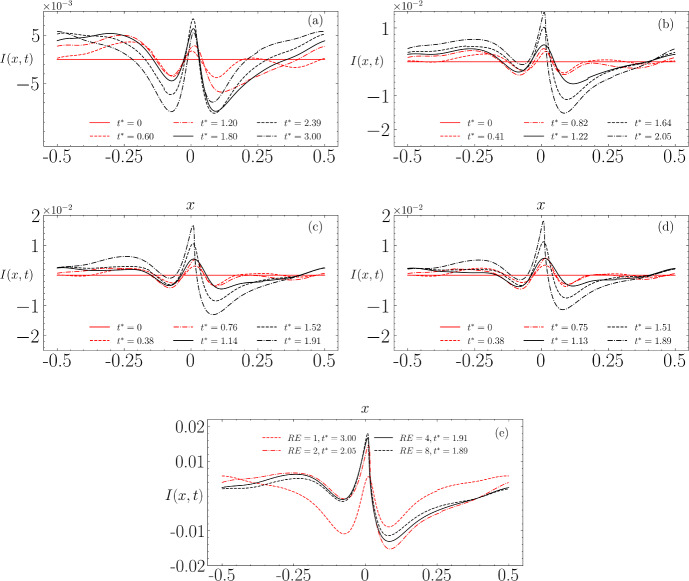
Evolution of instability using different Reynolds numbers, computed with fifth-order initial conditions and $\epsilon = 0.39$: **a** Re $=1\times 10^4$, **b** Re $=2\times 10^4$, **c** Re $=4\times 10^4$, **d** Re $=8\times 10^4$. **e** shows the instabilities at the final time for comparison

Figure 20 displays the corresponding instabilities for each wave profile. The instability for Re $=1\times 10^4$ initially develops into a dipole-like shape, observable in plots between $t^*=0$ and $t^*=2.39$, before settling into an almost symmetrical shape. Conversely, instabilities at higher Reynolds numbers (Re $=2\times 10^4; 4\times 10^4; 8\times 10^4$) progressively evolve towards a dipole-like shape, leading to wave breaking. Specifically, wave breaking occurs at $t^*=2.05$ for Re $=2\times 10^4$, $t^*=1.91$ for Re $=4\times 10^4$, and $t^*=1.89$ for Re $=8\times 10^4$.

When analysed collectively, these cases exhibit qualitative differences. Geometrically, a decrease in viscosity corresponds to an increase in the peak of the dipole and a decrease in its minima. Temporally, a decrease in viscosity leads to a faster development of the dipole-like instability that precedes wave breaking.

In these simulations, viscous dissipation serves as the primary mechanism of energy loss in the absence of wave breaking. The total energy decays as $E \propto e^{-\alpha t^*}$, where $\alpha$ is a decay coefficient that increases with viscosity [2], and confirmed in DNS studies [15]. Consequently, higher viscosity levels enhance dissipation, potentially preventing the instability from evolving into a dipole shape, as observed in the case with Re $=1\times 10^4$, while lower viscosity allows for less energy dissipation, resulting in faster development of the dipole-like instability.

### Effect of Surface Tension

Similarly to the previous section, we now investigate the effects of surface tension through the Bond number, $\text {Bo} = \Delta \rho g / {\gamma } k^2$. It is important to note that the initial conditions (4.4) and (4.5) do not include surface tension; however, its effect begins to manifest as the wave starts propagating. The Bond number is varied to take values of $\text {Bo} = 100$, $\text {Bo} = 500$, $\text {Bo} = 1000$, and $\text {Bo} = 2000$, corresponding respectively to physical wavelengths of $\lambda \sim 18~\textrm{cm}$, $\lambda \sim 41~\textrm{cm}$, $\lambda \sim 58~\textrm{cm}$, and $\lambda \sim 82~\textrm{cm}$.

**Fig. 21:**
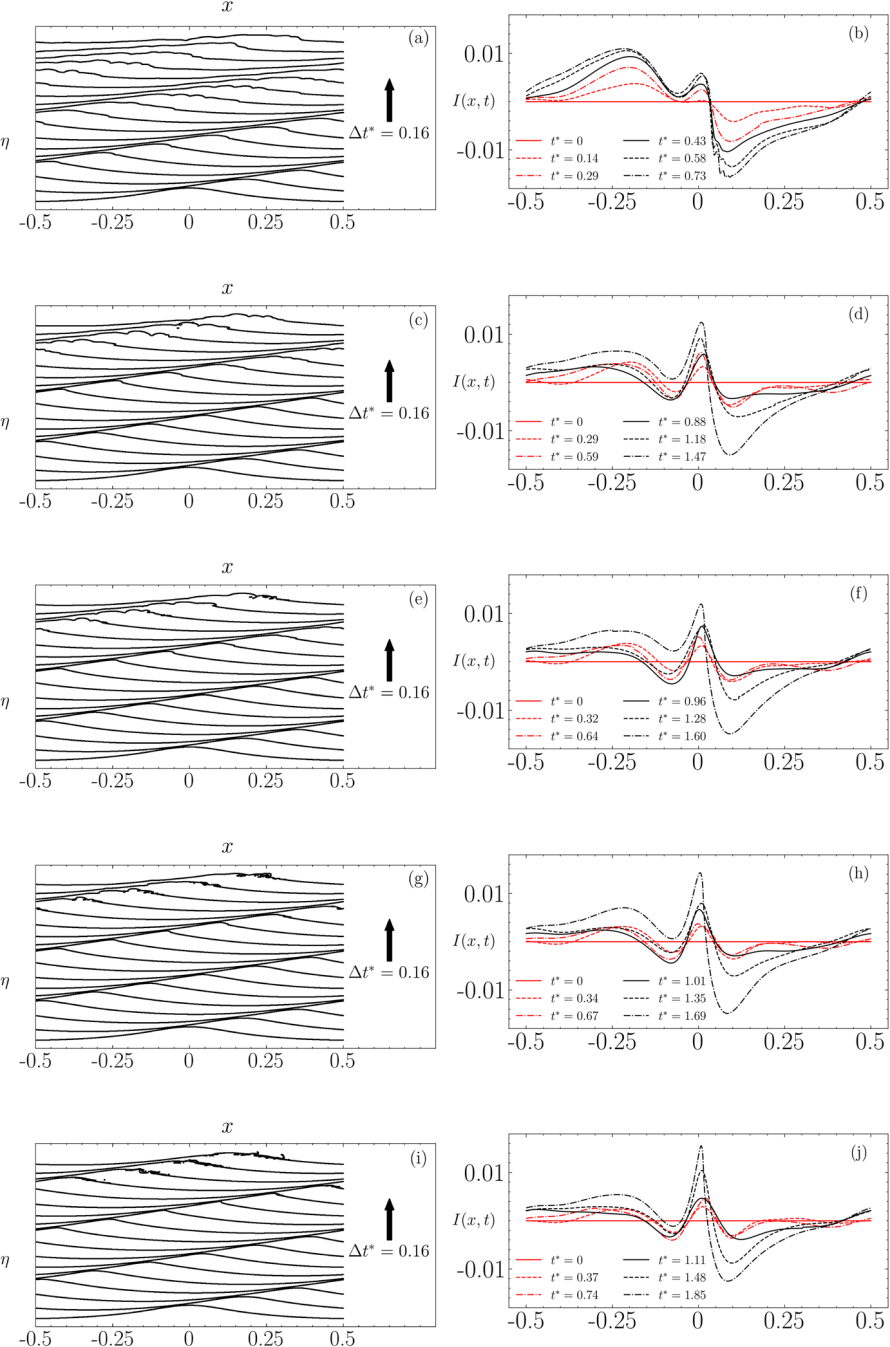
Evolution of the wave profile (left) and the corresponding computed instability $I(x,t)$ (right) at $\epsilon = 0.39$ for each value of the Bond number. **a**, **b** $\textrm{Bo} = 100$; **c**, **d** $\textrm{Bo} = 500$, **e**, **f** $\textrm{Bo} = 1000$, **g**, **h** $\textrm{Bo} = 2000$, and **i**, **j** No surface tension

In Fig. 21, panel a shows the breaking wave for $\mathrm{Bo =} 100$ characterised by a bulge trailing behind a toe at the crest, where surface tension is highest due to high curvature. Initially stationary relative to the carrier wave, parasitic capillaries are created. The toe then accelerates down the wave face, generating ripples that spread across the entire wave profile, with breaking occurring at $t^* = 0.73$. Parasitic capillary waves, first theoretically described by Ref. [40] and [12], were later confirmed by numerical studies such as [18, 21, 56, 42], and [15]. These studies highlighted their role in dissipating energy from the carrier wave through viscous dissipation or balancing the energy input from the wind. The gravity-capillary wave-breaking process observed here is consistent with the descriptions provided in these studies.

In panel (c) with $\textrm{Bo} = 500$, the wave breaking at $t^* = 1.47$ is similar to the previous case, but with less pronounced capillary waves, resulting in a spilling-type breaking. In panel (e) with $\textrm{Bo} = 1000$, reduced surface tension effects lead to an initially near-overturning crest breaking in a spilling manner at $t^* = 1.60$. The trend from parasitic capillaries and spilling breaking to a purely overturning microbreaker continues in panel (g) with $\textrm{Bo} = 2000$, where the downward motion is quickly followed by a small overturning near the crest. This trend is also observed in panel (i), where the absence of surface tension leads to the initiation of breaking by an overturning microbreaker.

Examining the development of the instability function $I(x,t)$ in relation to the breaking wave and the breaking onset time, we observe that, with the exception of panel (b) for $\textrm{Bo} = 100$, where breaking occurs without the instability forming a dipole-like shape, likely due to the emergence of parasitic capillaries, the instability function in panels (d), (f), and (h) (corresponding to $\textrm{Bo} = 500$, $\textrm{Bo} = 1000$, and $\textrm{Bo} = 2000$, respectively) shows a progression from an almost-symmetric shape to a dipole shape. This trend is also evident in the no surface tension case shown in panel (j).

**Fig. 22:**
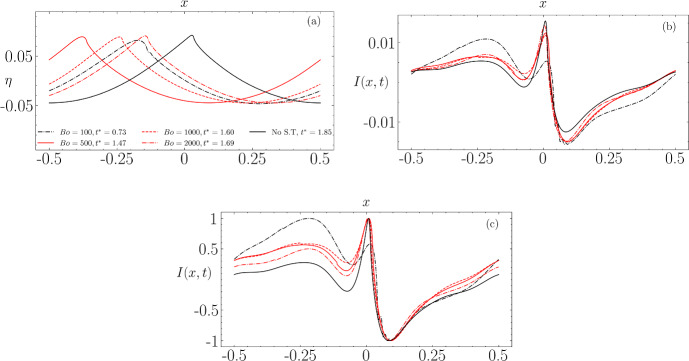
**a** Wave profile and **b** corresponding instability profile at the time of breaking. **c** Instability profile from (**b**) scaled to the range $[-1, 1]$ for comparison

In Fig. 22, panel (a) displays the wave profiles prior to breaking for each Bond number studied, alongside their corresponding instability functions at the same timestep for comparison. Panel (c) shows that, except for the $\textrm{Bo} = 100$ case, there are noticeable differences in the dipole-like shapes of the instability functions, particularly in the region where $x < 0$. The no surface tension case closely matches the theoretical representation of the dipole-like instability function, appearing closer to zero in this region. Among the tested values, the $\textrm{Bo} = 2000$ case most closely approximates the no surface tension scenario as we might expect.

Consequently, similar to the observations made in the previous section regarding the influence of viscosity through the physical wavelength, a comparable conclusion can be drawn here. When surface tension effects become significant, as in the case of capillary or short gravity waves, there appears to be no clear numerical evidence of the superharmonic instability, as theoretically described, playing a direct role in the breaking process.

## Concluding Remarks

The main result of this paper is to confirm the importance of the superharmonic instability of Stokes travelling waves as a mechanism for wave breaking in real-world conditions such as the open ocean. However, we have just scratched the surface of this problem. The most dramatic limitation is that the analysis here is for a two-dimensional slice of the ocean. What happens when the basic state is three dimensional? In work in progress, the authors are looking at the SH instability of short-crested Stokes waves. This class of waves is genuinely three-dimensional, yet steady in a moving frame, and can be studied with the same methodology: they are SH unstable for a region of parameter space, the eigenfunctions can be computed exactly (within numerical error), and Basilisk has a three-dimensional version.

Already in two dimension, there are questions about the role of surface tension. Linear stability calculations, discussed in §2, show that large surface tension can stabilise the SH instability. Numerical results of Dyachenko and Newell [18] show that the character of wave breaking changes with surface tension, and the experiments of Duncan et al. [19] indicate that surface tension appears to affect the jet of the overturning breaker, particularly when the wavelength is short, and indeed may prevent the wave from overturning. The shape of the unstable eigenfunction is also affected by surface tension (e.g. [13]). Deike et al. [15] argue that the presence of capillarity increases the energy dissipation below the breaking threshold.

Finite, and particularly small, depth will affect the SH instability and subsequent wave breaking. Kataoka [33] shows that mass flux plays no role in infinite depth, but becomes increasingly important in finite depth.

The SVD algorithm for filtering the perturbation will need to be extended to 3D. This appears to be possible in principle, and will be useful for extracting the geometric structure of the unstable perturbations. This will also enable the analysis to follow the evolution over time of the unstable perturbation until breaking and post-breaking, since the primary results suggest that the three-dimensional extracted instability exhibits a much more complex structure, and that the micro-breaking, occurring near the crest region as a result of this instability, does not significantly affect the evolution of the remaining components.

## Data Availability

No datasets were generated or analysed during the current study.

## A Jacobian Matrix for Basic Flow Solver

In this appendix, we identify how to solve for the components of the Jacobian matrix, $\dfrac{\partial F_m}{\partial A_j}$, $\dfrac{\partial F_m}{\partial c}$, $\dfrac{\partial W}{\partial A_j}$, $\dfrac{\partial W}{\partial c}$, that arise in the algorithm in Sect. 3, and write these down in terms of components. Intrinsically, we find

A-1 $$\begin{aligned} \frac{\partial F_m}{\partial A_j}=\int _0^\pi \left[ \frac{\partial Q}{\partial A_j}+\frac{\partial \phi }{\partial z}\frac{\partial \eta }{\partial A_j}\right] \sin (mX)\textrm{d}X, \end{aligned}$$

but as noted by Okamura [45], we do not have information about $\dfrac{\partial \eta }{\partial A_j}$, and hence we need to eliminate it. We do this by noting that

$$\begin{aligned} \frac{\partial P}{\partial A_j}+\frac{\partial P}{\partial z}\frac{\partial \eta }{\partial A_j}=0\,, \end{aligned}$$

and so

A-2 $$\begin{aligned} \frac{\partial F_m}{\partial A_j}=\int _0^\pi \left[ \frac{\partial Q}{\partial A_j}-\frac{\partial \phi }{\partial z} \frac{\frac{\partial P}{\partial A_j}}{\frac{\partial P}{\partial z}}\right] \sin (mX)\,\textrm{d}X. \end{aligned}$$

Similarly, using this approach, we can find that

A-3 $$\begin{aligned} \frac{\partial F_m}{\partial c}= &   \int _0^\pi \left[ \frac{\partial Q}{\partial c}-\frac{\partial \phi }{\partial z} \frac{\frac{\partial P}{\partial c}}{\frac{\partial P}{\partial z}}\right] \sin (mX)\,\textrm{d}X, \end{aligned}$$

A-4 $$\begin{aligned} \frac{\partial W}{\partial A_j}= &   \left. \frac{\frac{\partial P}{\partial A_j}}{\frac{\partial P}{\partial z}}\right| _{x=0}-\left. \frac{\frac{\partial P}{\partial A_j}}{\frac{\partial P}{\partial z}}\right| _{x=\pi }, \end{aligned}$$

A-5 $$\begin{aligned} \frac{\partial W}{\partial c}= &   \left. \frac{\frac{\partial P}{\partial c}}{\frac{\partial P}{\partial z}}\right| _{x=0}-\left. \frac{\frac{\partial P}{\partial c}}{\frac{\partial P}{\partial z}}\right| _{x=\pi }. \end{aligned}$$

In practical terms, the important relations in calculating the Jacobian are

$$\begin{aligned} \frac{\partial P}{\partial z}= &   -c\phi _{Xz}+\phi _X\phi _{Xz}+\phi _z\phi _{zz}+1,\\ \frac{\partial P}{\partial A_j}= &   j\left[ (\phi _X-c)\cos (jX)+\phi _z\sin (jX)\right] e^{jz},\\ \frac{\partial P}{\partial c}= &   -\phi _X,\\ \frac{\partial Q}{\partial z}= &   c^2\phi _{XXz}+\phi _{Xz}\left[ -2c\phi _{XX}+\phi _X\phi _{XX}+\phi _z\phi _{Xz}\right] , \\  &   +\phi _{X}\left[ -2c\phi _{XXz}+\phi _{Xz}\phi _{XX}+\phi _X\phi _{XXz}+\phi _{zz}\phi _{Xz}+\phi _z\phi _{Xzz}\right] \\  &   +\phi _{zz}\left[ -2c\phi _{Xz}+\phi _X\phi _{Xz}+\phi _z\phi _{zz}+1\right] \\  &   +\phi _z\left[ -2c\phi _{Xzz}+\phi _{Xz}^2+\phi _X\phi _{Xzz}+\phi _{zz}^2+\phi _z\phi _{zzz}\right] ,\\ \frac{\partial Q}{\partial A_j}= &   2j\left[ -c\phi _{XX}+\phi _X\phi _{XX}+\phi _z\phi _{Xz}+j\phi _X\phi _z-c j\phi _z\right] \cos (jX)e^{jz}\\  &   +j\left[ -jc^2+\left[ j\phi _z^2-2c\phi _{Xz}+\phi _X\phi _{Xz}+2\phi _z\phi _{zz}+1\right] \right. \\  &   \left. +\phi _X\left[ 2c j-j\phi _X+\phi _{Xz}\right] \right] \sin (jX)e^{jz},\\ \text{ and }\\ \frac{\partial Q}{\partial c}= &   2c\phi _{XX}-2\left[ \phi _X\phi _{XX}+\phi _z\phi _{Xz}\right] . \end{aligned}$$
